# Understanding the Effects of Conductive Polymer Electrode Coating on Recorded Neural Signals

**DOI:** 10.1002/adhm.202503893

**Published:** 2026-02-21

**Authors:** Karthik Sridhar, Judith Evers, Alexandre Trotier, Manus Biggs, Madeleine M. Lowery

**Affiliations:** ^1^ School of Electrical and Electronic Engineering University College Dublin Dublin Ireland; ^2^ Research Ireland CÚRAM Research Centre For Medical Devices University of Galway Ireland; ^3^ School of Veterinary Medicine University College Dublin Dublin Ireland; ^4^ Paris Brain Institute (ICM) Inserm U1127, CNRS UMR 7225, Sorbonne University Hôpital de la Pitié‐Salpêtrière Paris 75013 France

**Keywords:** PEDOT:PTS, amplifier impedance, electrode impedance, electrode‐tissue interface, finite element model, local field potential, neural recoding and stimulation

## Abstract

Conductive polymer coatings have been extensively explored as a means of improving the quality of neural signals recorded with chronically implanted electrodes. They offer enhanced biocompatibility along with reduced electrode impedance and are reported to improve signal‐to‐noise ratio and signal amplitude. The mechanisms by which poly(3,4ethylenedioxythiophene) (PEDOT) and its derivatives enhance the quality of neural signals recorded in vivo, however, remain unclear. Here, a computational model of PEDOT:PTS (polythiophenesulfonyl chloride) coated neural recording electrodes is used to understand how the different properties of conductive electrode coatings influence local field potentials recorded in vivo. Impedance, histology and electrophysiology data were obtained from coated and uncoated microelectrodes chronically implanted in the rat basal ganglia and incorporated in the model. Together the simulation and experimental results indicate that improvements in signal quality with PEDOT:PTS coated electrodes are driven by greater neural proximity to the electrode, facilitated by reduced peri‐electrode gliosis. Reductions in thermal noise with decreasing electrode impedance further contributed to a higher signal‐to‐noise ratio for PEDOT:PTS coated electrodes. Finally, the results demonstrate that, provided amplifier input impedance requirements are satisfied, the enhanced recording capability of polymer coated electrodes compared to uncoated electrodes is due primarily to improved biocompatibility rather than reduced electrode impedance.

## Graphical abstract

Conductive polymer coatings are widely explored to improve the quality of signals recorded with chronically implanted neural electrodes, offering enhanced biocompatibility along with reduced electrode impedance. Combining computational modelling and data from PEDOT:PTS coated and PtIr electrodes in rats, we show that improvements in signal quality with polymer coated electrodes are driven primarily by improved biocompatibility rather than reduced electrode impedance.

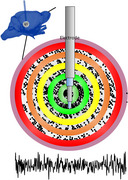

## 1 Introduction

The development of chronically implantable neural interfaces which provide high‐quality, stable, long‐term recording of neural activity remains a major challenge for clinical applications of brain‐machine interfaces. Brain‐machine interfaces that enable direct communication between the brain and external devices offer the potential to restore the ability to communicate to patients living with severe neurological disorders including high‐level spinal cord injury, stroke and amyotrophic lateral sclerosis [1]. Incorporating sensing capability within neuromodulation devices facilitates the implementation of closed‐loop or adaptive stimulation which has the potential to improve therapeutic efficacy [2]. These types of neuroprostheses rely on chronically implanted metal electrodes, typically platinum or its alloys. Driven by a need for stable, high quality neural interfaces, conductive polymer electrode coatings have become increasingly popular as a way to enhance the quality of neural recordings. A wide range of conductive polymer coatings have been developed, all of which reduce electrode impedance and enhance biocompatibility [3, 4]. However, the mechanisms by which conductive polymer coatings enhance the quality of neural signals recorded in vivo, and the extent to which they do so, remain unclear.

Experiments conducted using neural recording electrodes have established that conductive polymer coatings such as poly(3,4ethylenedioxythiophene) (PEDOT) on metal electrodes dramatically reduce electrode‐tissue impedance, which is associated with improved signal‐to‐noise ratio (SNR), and provide stable electrochemical performance [5, 6, 7]. In addition to enhancing the SNR and electrochemical stability, PEDOT‐based conductive coatings aid the growth of axons and stabilize the peri‐electrode space by reducing gliosis [8, 9, 10]. However, while reduced impedance and higher SNR have been observed for conductive polymer coated electrodes [5], the combined effect of changes in impedance, gliosis and neuron density on the signal recorded at the electrode remains poorly understood. Furthermore, separating the relative contribution of these different factors to understand the underlying mechanisms in vivo is challenging. The results of experimental studies on the effect of electrode impedance have been inconclusive or contradictory, with both substantial and negligible effects of electrode impedance on the quality of recorded neural signals reported [6, 11, 12, 13]. Reduced signal quality from chronically implanted neural electrodes is often attributed to the high impedance of the encapsulation layer that forms around the electrode during gliosis [14, 15, 16, 17, 18]. An alternative possibility is that the electrode is physically isolated from nearby neurons by the encapsulation layer, which lowers the density of surrounding neurons, consequently degrading signal quality [17, 19]. In addition to these factors, increased impedance and occlusion of the electrode surface due to protein adsorption, or biofouling, may contribute to degradation of the quality of neural signals recorded with implanted electrodes [15, 17, 20]. This effect can be mitigated by the application of a current or voltage which rejuvenates the electrode interface and can lead to differences in the electrode‐tissue impedance of recording and stimulation electrodes [21]. Understanding how the electrode interface changes with stimulation and the potential impact of this on neural recording is increasingly important as the simultaneous use or cycling of electrodes for stimulation and sensing becomes more widespread with adaptive stimulation paradigms [2]. Intrinsically recorded noise, the largest component of which is thermal noise, also affects the SNR and is positively correlated with electrode impedance [22], while at very high electrode impedances voltage division across the amplifier and electrode interface can reduce the input voltage at the amplifier. This effect becomes negligible once the electrode impedance is sufficiently low relative to that of the amplifier but can be a challenge in devices with high impedance microelectrodes [11].

To understand the contribution of the different factors which influence neural signals recorded using conductive polymer coatings, we developed a computational model of neural signals recorded with poly(3,4ethylenedioxythiophene)/ polythiophenesulfonyl chloride (PEDOT:PTS) coated and platinum iridium (PtIr) electrodes. The model was developed using electrode impedance and histology data experimentally recorded using microelectrodes chronically implanted in the rat basal ganglia for 8 weeks. PEDOT:PTS exhibits low impedance at biologically relevant frequencies and has a higher charge storage capacity than other PEDOT derivatives such as PEDOT:PSS [23, 24]. Polythiophenesulfonyl chloride (PTS) binds tightly to PEDOT giving it better long‐term electrochemical and structural stability with less delamination or swelling than polystyrene sulfonate (PSS) [25]. The properties of the electrical double layer interface and histological properties of the surrounding glial tissue were characterised and local field potential (LFP) signals were recorded for the PEDOT:PTS coated and uncoated PtIr electrodes. Impedance data were recorded from both standard chronically implanted PtIr recording electrodes and PtIr electrodes in which electrode impedance was reduced by the application of a current which reduced biofouling due to protein absorption [26]. A finite element (FE) model of PEDOT:PTS coated and PtIr microelectrodes was developed incorporating the electrode‐tissue interface, electrode double layer impedance and histological encapsulation tissue properties estimated experimentally. The computational model was then used to simulate the effect of polymer coating on LFP signals recorded using microelectrodes in the rat brain.

The electrical double layer properties of PEDOT:PTS coated and pristine PtIr electrodes were first estimated and incorporated within the model to examine the influence of electrode impedance on signal amplitude as amplifier input impedance was varied. Reduced signal amplitude due to voltage division was observed when the electrode impedance was insufficiently low relative to the amplifier impedance. The effect of electrode impedance on LFP signal amplitude and the detection volume of each electrode was then examined. Simulated LFP signals were compared for chronically implanted PEDOT:PTS coated electrodes, standard PtIr electrodes and PtIr electrodes in which electrode impedance was reduced through the application of a current which reduces protein absorption. The results confirmed that electrode impedance alone did not affect the amplitude of the detected neural signal or electrode detection volume, provided the amplifier impedance was sufficiently high. Next, the influence of the encapsulating glial tissue was examined. The histological properties of the tissue in the peri‐electrode space, characterised experimentally, were incorporated in the computational model along with thermal noise at the electrode. The amplitude of the detected LFP signal and the SNR were estimated as encapsulation tissue properties and neural density in the region surrounding the electrode were varied. The results illustrate higher signal amplitude and SNR for the PEDOT:PTS coated electrodes due to a reduction in gliosis and closer proximity of neurons to the electrode and reduced thermal noise. Finally, LFP data recorded using chronically implanted microelectrodes in the rat brain were compared between coated and uncoated electrodes. The results demonstrated higher signal amplitude and SNR at 8 weeks post‐surgery, as predicted by the computational models. Together the results provide insight into the mechanisms by which polymer coatings improve the quality of recorded neural signals and identify enhanced biocompatibility and closer neural proximity to the electrode as the primary determinant of enhanced signal quality with polymer coated neural recording electrodes.

## 2 Results

### 2.1 Impedance of PEDOT:PTS Coated and PtIr Electrodes

#### 2.1.1 In Vivo Electrode Impedance

**FIGURE 1:**
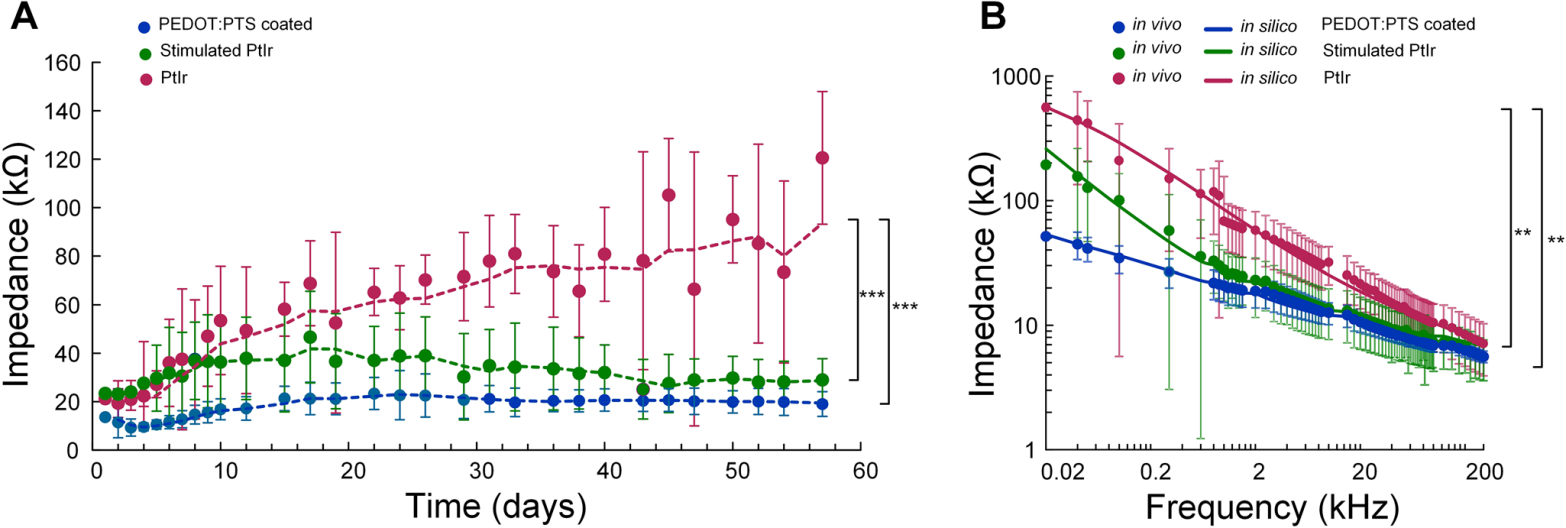
Impedance of PEDOT:PTS coated and PtIr electrodes recorded in vivo. A. Impedance of PEDOT:PTS coated (*N* = 6), uncoated (*N* = 7) and uncoated stimulated (N = 6) PtIr electrodes at 1 kHz recorded over 56 days in vivo (two‐way repeated‐measures ANOVA: Coating: F(2,464) = 16.99, *p* = 0.0001, Time: F(29 464) = 19.65, *p* < 0.0001, Interaction: F(58 464) = 3.37, *p* < 0.0001, Bonferroni post‐test). B. Impedance spectra for each electrode recorded in vivo at 8 weeks post‐surgery (two‐way repeated‐measures ANOVA: Coating: F(2,1264) = 11.00, *p* = 0.001, Frequency: F(79,1264) = 16.91, *p* < 0.0001, Interaction: F(158,1264) = 4.79, *p* < 0.0001, Bonferroni post‐test). Impedance data measured in silico in the FE model are also presented. Mean ± SD presented for experimental data. Impedance spectra of the unstimulated and stimulated PtIr electrode are obtained from Evers et al., [21]. ^***^ *p* < 0.001, ^**^_< 0.01.

The frequency‐dependent electrode impedance of chronically implanted PEDOT:PTS coated and PtIr electrodes (SNEX‐100 concentric bipolar tapered electrode, Microprobes, Gaithersburg, USA), recorded over 8 weeks was first established Figure 1. The electrodes had a recording tip of Ø 100 µm with an active surface area of 0.078 mm^2^ and a stainless‐steel reference contact with a surface area of 0.34 mm^2^, positioned 0.5 mm apart. Electrode impedance was lower in the PEDOT:PTS coated electrodes relative to the impedance of pristine PtIr electrodes in both the unstimulated and stimulated state. Stimulated PtIr electrodes had received electrical stimulation for approximately 7 h/day from 3 days to 8 weeks post‐operative, with a 130 Hz charge balanced pulse of pulse duration 60 µs and pulse amplitude of 80–100 µA. The pulse amplitude was determined based on the threshold for stimulation induced side effects in the individual rats [21]. At implantation, electrode impedance at 1 kHz was 13.6 kΩ (± 2.8 kΩ) in PEDOT:PTS, 21.2 kΩ (± 2.4 kΩ) in unstimulated PtIr and 23.4 kΩ (± 5.5 kΩ) in stimulated PtIr electrodes. While the impedance of the PtIr electrodes increased progressively during the first 3 weeks following implantation, impedance of the PEDOT:PTS coated electrodes remained low and was stable from 15 days onward (Figure 1A). At 8 weeks, the impedance at 1 kHz was 20.3 kΩ (± 5.3 kΩ) in PEDOT:PTS, 61.9 kΩ (± 27.1 kΩ) in unstimulated PtIr and 25.5 kΩ (± 9.6 kΩ) in stimulated PtIr electrodes. Significant differences between electrode types were observed from 10 days onward (two‐way repeated‐measures ANOVA: Coating: F(2,464) = 16.99, *p* = 0.0001, Time: F(29,464) = 19.65, *p* < 0.0001, Interaction: F(58,464) = 3.37, *p* < 0.0001, Bonferroni post‐test). The impedance of the PEDOT:PTS coated electrode was substantially lower than that of the PtIr, both stimulated and unstimulated, at frequencies up to 10 kHz Figure 1B (two‐way repeated‐measures ANOVA: Coating: F(2,1264) = 11.00, *p* = 0.001, Frequency: F(79 1264) = 16.91, *p* < 0.0001, Interaction: F(158 1264) = 4.79, *p* < 0.0001, Bonferroni post‐test).

#### 2.1.2 Estimation of Electrode Electrical Double Layer Properties

A difference in charge carriers in the electrode and surrounding electrolyte results in the formation at the electrode‐tissue interface of a high impedance electrical double layer (EDL) which can be represented as the parallel combination of a pseudocapacitive constant phase angle element, *Z_cpe_* , and a charge transfer resistance, *R_ct_* [27]. The double layer properties of the PEDOT:PTS and PtIr electrodes estimated in saline solution in vitro are presented in Table 1. The EDL of the PEDOT:PTS and PtIr electrodes were incorporated using the thin layer approximation [28] into a 3D heterogenous FE model of the rat brain and SNEX‐100 microelectrode, and the impedance at the electrode was measured in the model. As the estimated value of *R_ct_* exceeded 1 MΩ for all conditions (unstimulated, stimulated, and coated electrodes), Faradaic charge transfer was assumed to be zero and the EDL was represented by *Z_cpe_* in subsequent models. The impedance spectra obtained from the model agreed well with the impedance spectra recorded experimentally in vivo for each electrode, with a mean difference between simulated and experimental values of 6.7% ± 5.2%, Figure 1B. Using the electrode impedance measured in vivo, the root mean square (RMS) amplitude of the thermal noise in the frequency range from 100 Hz to 8 kHz was then estimated. The predicted RMS value of the thermal noise for the PEDOT:PTS electrode was 1.67 µV, while that of the PtIr and stimulated PtIr electrodes was 8.78 and 4.9 µV, respectively Table 1.

**TABLE 1:** Estimated equivalent circuit parameters of the electrical double layer of the PEDOT:PTS coated and PtIr electrodes characterized in 0.9% saline solution at room temperature. The parameters for the PtIr electrodes are based on the data reported in Evers et al., [21]. The estimated amplitude of the thermal noise for each electrode is also presented. The electrical double layer is represented as the parallel combination of a pseudocapacitive constant phase angle element, *Z_cpe_* , and a charge transfer resistance, *R_ct_*, where *Z_cpe_* =  *K*(*j*ω)^−β^.

Electrode	K (Ω m^2^ s^−β^)	Beta(β)	Thermal noise RMS amplitude
PEDOT:PTS coated electrode	0.031	0.60	1.67 µV
PtIr electrode	2.14	0.67	8.78 µV
PtIr electrode (stimulated)	1.42	0.85	4.9 µV

#### 2.1.3 Combined Effect of Amplifier and Electrode Impedance on Signal Amplitude

**FIGURE 2:**
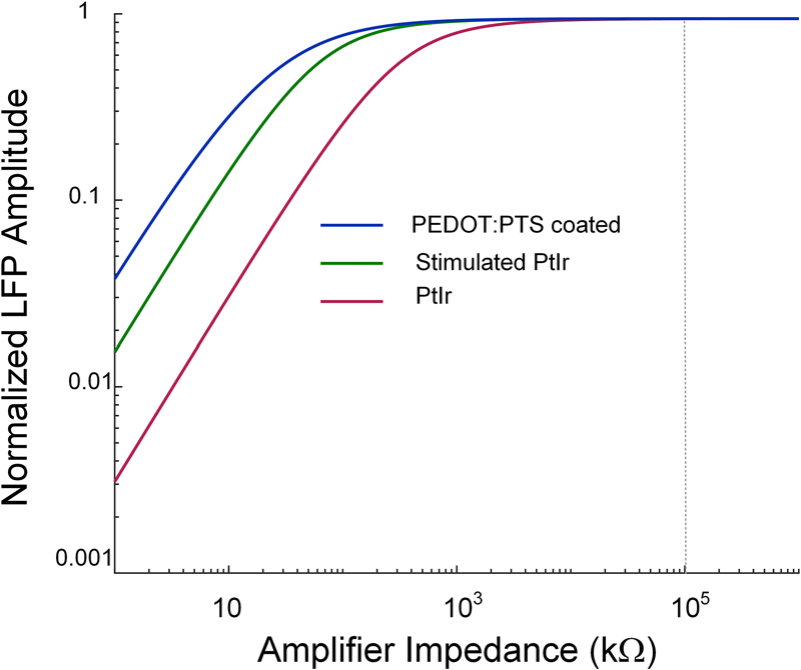
Effect of amplifier input impedance on the root mean square (RMS) amplitude of simulated local field potentials (LFPs). The amplitude of the LFP signal has been normalized with respect to the value detected with an infinitely high amplifier input impedance. The results confirm that the amplifier impedance of 100 MΩ (NL844, Digitimer Ltd, Hertfordshire) used for the experimental recordings (dashed line) was sufficiently high to avoid signal attenuation due to voltage division for all electrodes examined.

The effect of amplifier input impedance on the amplitude of the detected LFP signal was then examined using the model for the three electrodes. When the input impedance of the amplifier was sufficiently high relative to the electrode impedance, neither amplifier nor electrode impedance altered the signal detected at the amplifier Figure 2. As amplifier impedance decreased relative to electrode impedance, the amplitude of the signal detected at the amplifier input decreased due to voltage division across the amplifier and double layer. The amplifier impedance at which no discernible effect on the detected voltage was observed decreased with electrode impedance and was lower for coated electrodes than for uncoated electrodes, and for stimulated electrodes when compared with unstimulated electrodes Figure 2. The simulation results confirm that the input impedance of the amplifier used for the in vivo experiments (100 MΩ) was sufficiently high to ensure that the voltage dropped across the electrode interface was negligible for the electrodes examined.

#### 2.1.4 Effect of Electrode Impedance on Signal Amplitude and Electrode Detection Volume

**FIGURE 3:**
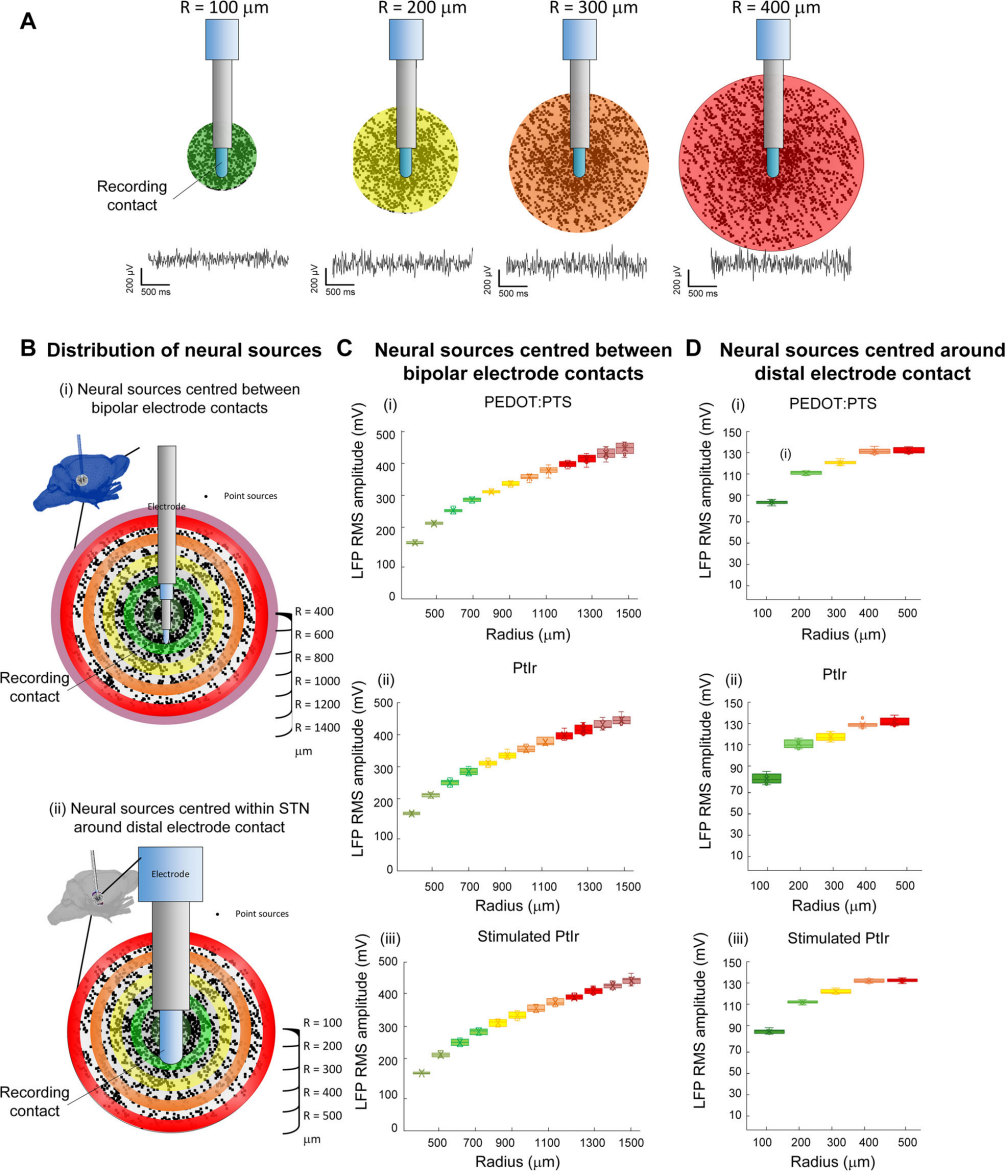
Effect of electrode impedance on local field potential (LFP) amplitude and electrode detection volume. (A) Representative examples of source distributions and simulated LFP signals for point current sources distributed within spheres of radius 100, 200, 300, and 400 µm centered on the distal recording electrode. Data were simulated for a homogeneous volume conductor with uniform weighting of current sources. (B) (i) Distribution of synaptic point current sources within a sphere ranging in radius from (i) 400 to 1500 µm centered midway between the recording electrodes and (ii) 100 to 500 µm centered on the distal recording electrode. (C) Root mean square (RMS) amplitude of the simulated LPF signals for PEDOT:PTS coated, unstimulated and stimulated PtIr electrodes for sources distributed as in B(i) as the volume containing active sources was progressively increased. The electrode impedance had no significant effect on LFP signal amplitude or on the detection volume of the electrode (two‐way repeated‐measures ANOVA, Radius: F(11,297) = 7856, *p* < 0.0001, Electrode: F(2,297) = 0.95, *p* = 0.40, Interaction: F(22,297) = 0.80, *p* = 0.73). (D) RMS amplitude of the simulated LPF signals for PEDOT:PTS coated, unstimulated and stimulated PtIr electrodes as the volume containing active sources was progressively increased for sources distributed as in B(ii). Electrode impedance had no significant effect on LFP signal amplitude at distances > 100 µm or on the detection volume of the electrode (two‐way repeated‐measures ANOVA and Bonferroni post‐test, Radius: F(4,108) = 1915, *p* < 0.0001, Electrode: F(2,108) = 7.43, *p* = 0.0027, Interaction: F(8,108) = 2.69, *p* = 0.01). The results from 10 simulations with randomly distributed neuronal sources are presented.

The effect of PEDOT:PTS polymer coating on LFP amplitude and the electrode detection volume was then examined using the computational model. To estimate the detection volume of the electrode, RMS amplitude was estimated for simulated bipolar LFP signals as the volume of tissue containing active sources was progressively increased Figure 3A. The amplitude of the LFP signal increased with the volume of tissue containing active sources until it reached a point beyond which sources were sufficiently far from the electrode that their contribution was negligible. The detection volume of the electrode was defined as the volume of active tissue surrounding the electrode for which the RMS amplitude of the detected LFP signal was equal to 95% of the amplitude of the signal detected with all surrounding sources activated.

The amplitude of the detected LFP signal was first estimated as the radius of a sphere around the electrode containing active sources was progressively increased from 400 to 1500 µm. The sphere was centered mid‐way between the bipolar electrode contacts which were separated by an interelectrode distance of 0.5 mm Figure 3C. LFP RMS amplitude increased progressively as the volume of active tissue was increased up to a radius of approximately 1400 µm for all electrode interfaces, Figure 3. The LFP amplitude increased by less than 4% when the radius containing active sources was further increased from 1400 to 1500 µm; the radius of the electrode LFP detection volume was estimated to be 1348.9 ± 25.2 µm, 1347.0 ± 36.6 µm and 1359.3.9 ± 31.7 µm for the coated, PtIr and stimulated PtIr electrodes, respectively. ANOVA confirmed that there was no significant effect of electrode impedance on the volume of tissue activated (F(11 108) = 0.49, *p* = 0.62).

The amplitude of the detected LFP signals was similarly unaffected by electrode impedance. The RMS amplitude of the simulated LFP signal from sources lying within 1500 µm of the electrode differed by less than 2% across the electrodes examined (438.13 ± 15.76 µV for coated electrodes, 445.67 ± 12.33 µV for unstimulated uncoated electrodes and 443.75 ± 10.83 µV for stimulated electrodes). A two‐way repeated‐measures ANOVA confirmed that while LFP amplitude increased as the volume containing active sources increased, there was no effect of electrode impedance on LFP signal amplitude and no interaction between the radius of the volume containing active sources and electrode impedance (Radius: F(11 297) = 7856, *p* < 0.0001 η^2^ = 0.9966, Electrode: F(2297) = 0.95, *p* = 0.40, η^2^ = 0.1422, Interaction: F(22 297) = 0.80, *p* = 0.73, η^2^ = 0.0558).

To simulate a more physiological arrangement, similar to the experimental protocol where the electrode was located within the subthalamic nucleus (STN) of the basal ganglia, the simulations were repeated with the neural sources constrained within a sphere of radius 500 µm centered around the distal tip of the electrode Figure 3B(ii). The radius of the sphere containing active sources was progressively increased as before, and the amplitude of the LFP signal estimated. The RMS value of the LFP reached its maximum value when the sources lay within approximately 400 µm of the electrode Figure 3D. The radius of the electrode LFP detection volume was estimated to be 365.9 ± 36.1 µm, 379.3 ± 43.0 µm and 351.1 ± 25.3 µm for the coated, PtIr and stimulated PtIr electrodes, respectively, with no significant effect of electrode impedance (F(4,46) = 1.03, *p* = 0.37). Electrode impedance similarly had no significant effect on LFP amplitude, with less than a 2% variation in amplitude across electrodes, Figure 3C. For sources located within 400 µm of the electrode, the LFP RMS amplitude was 132.32 ± 2.25 µV for coated electrodes, 131.07 ± 2.42 µV for unstimulated uncoated electrodes and 132.56 ± 1.49 µV for stimulated electrodes. LFP amplitude increased with increasing volume of active tissue, however, electrode impedance had no significant effect on LFP signal amplitude at distances > 100 µm and there was no significant interaction between electrode impedance and the radius of the volume containing active sources (two‐way repeated‐measures ANOVA and Bonferroni post‐test, Radius: F(4,108) = 1915, *p* < 0.0001, η^2^ = 0.9861, Electrode: F(2,108) = 7.43, *p* = 0.0027, η^2^ = 0.1411, Interaction: F(8,108) = 2.69, *p* = 0.01, η^2^ = 0.1659).

**FIGURE 4:**
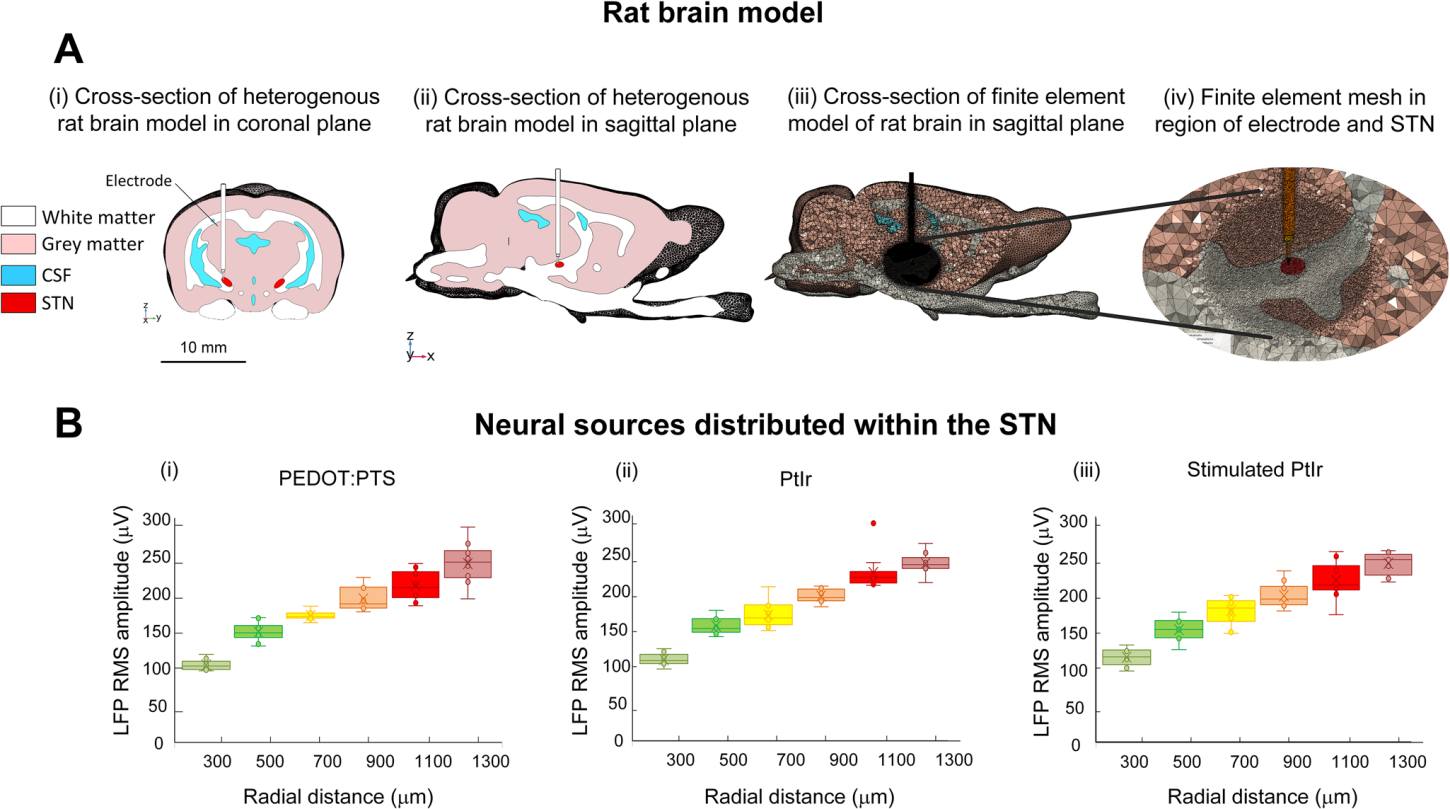
Effect of electrode impedance on local field potential (LFP) amplitude and electrode detection volume in a heterogeneous model of the rat brain with non‐uniform weighting of current sources within the subthalamic nucleus (STN). A. Cross‐section of heterogeneous rat brain model in (i) coronal and (ii) sagittal planes. The finite element mesh is shown in (iii) for the cross‐section in the sagittal plane and in (iv) for the region immediately surrounding the electrode. B. The root mean square (RMS) amplitude of the LFP signal as the radial distance between the electrode and the outer boundary of the region within the STN containing active sources was progressively increased from 300 to 1300 µm for (i) coated, (ii) PtIr and (iii) stimulated PtIr electrodes. Electrode impedance had no significant effect on LFP signal amplitude or on the detection volume of the electrode (two‐way repeated‐measures ANOVA and Bonferroni post‐test, Radial distance: F(5,162) = 248.2. *p* < 0.0001, η^2^ = 0.8846, Electrode: F(2,162) = 1.71, *p* = 0.1843, η^2^ = 0.0207, *p* = 0.40, Interaction: F(10.162) = 0.5231, *p* = 0.8721, η^2^ = 0.0313). The results from 10 simulations with randomly distributed neuronal sources are presented.

In the examples presented in Figure 3, the tissue surrounding the electrode was assumed to be homogeneous grey matter and neural sources of equal strength were randomly distributed within a spherical volume. To confirm the effect of electrode impedance in a more complex inhomogeneous model with sources distributed within the anatomical boundaries of the STN and a distributed weighting of current sources, a heterogeneous model of the brain was developed using image segmentation of Waxholm Space atlas of the Sprague Dawley rat Brain (WSSD)^)22^. Segmented masks of the grey matter, white matter and cerebrospinal fluid (CSF) were converted to a geometric model using Simpleware ScanIP (Synopsys, USA). The microelectrode was positioned within the STN with the aid of WSSD atlas as shown in Figure 4A. Sources were randomly distributed such that the total number of sources within the STN was 3000 and the strength of the current sources was randomly distributed according to a normal distribution with a mean weight of 0.5 and standard deviation of 0.15. The distance between the electrode and outer boundary of the volume containing active sources was increased progressively from 300 to 1300 mm and the RMS amplitude of the LFP signal detected at the electrode was estimated. As with the homogeneous model, the amplitude of the LFP signal increased as the volume of the active tissue increased and the impedance of the electrode had no significant effect on the LFP amplitude or detection volume, Figure 4B, (two‐way repeated‐measures ANOVA and Bonferroni post‐test, Radial distance: F(5,162) = 248.2. *p* < 0.0001, η^2^ = 0.8846, Electrode: F(2,162) = 1.71, *p* = 0.1843, η^2^ = 0.0207, *p* = 0.40, Interaction: F(10.162) = 0.5231, *p* = 0.8721, η^2^ = 0.0313).

### 2.2 Effect of PEDOT:PTS Coating on Peri‐Electrode Inflammation and Encapsulation

#### 2.2.1 Histological Characterization of Glial Response and Neural Distribution

**FIGURE 5:**
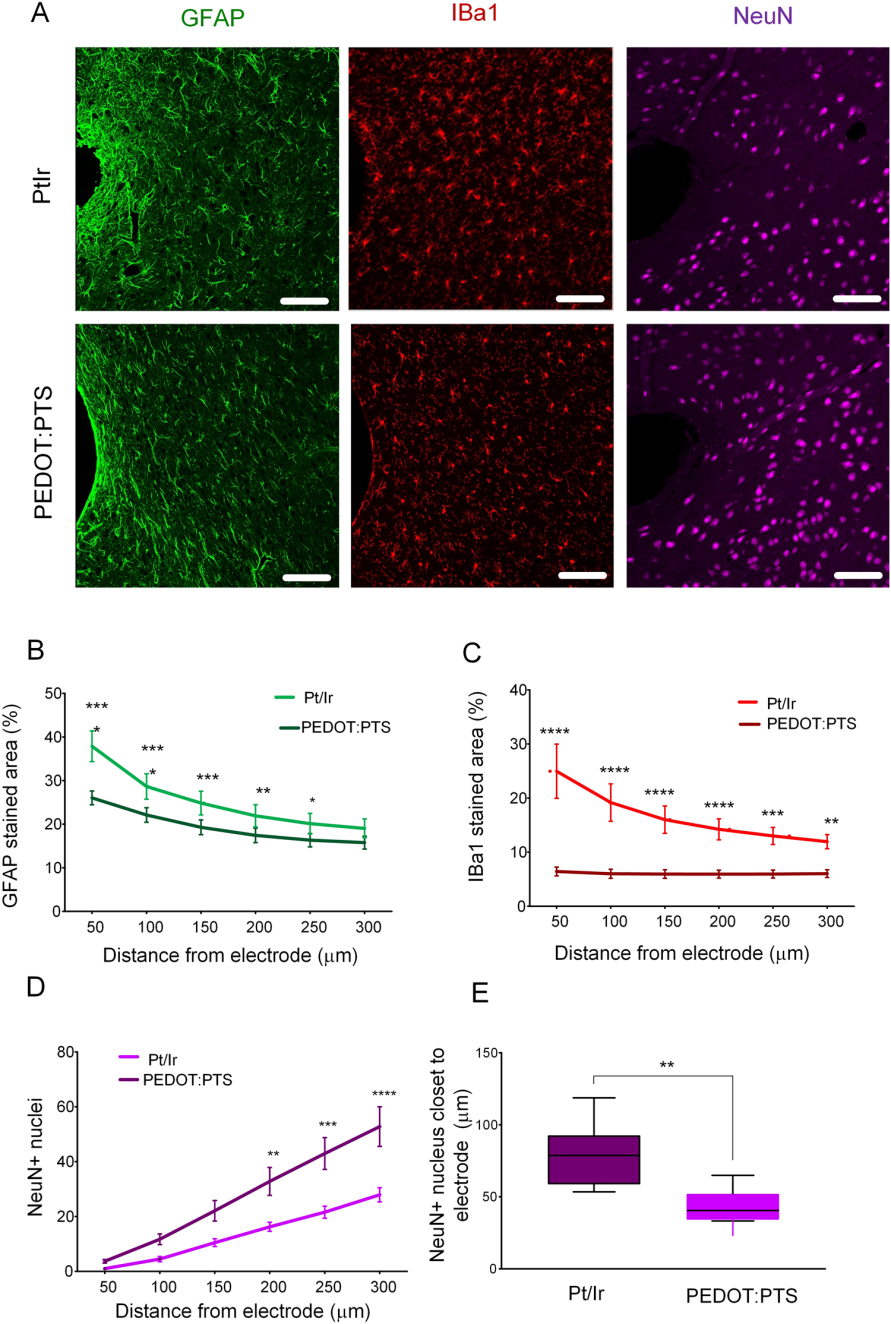
Foreign body response for PEDOT:PTS coated and PtIr electrodes. (A) Representative immunofluorescence images of brain tissues implanted with tapered PtIr control (*N* = 6), and PEDOT:PTS coated electrodes (scale bar = 100 µm). (B) Staining area of the astrocytic protein glial fibrillary acidic protein (GFAP) (two‐way repeated‐measures ANOVA with Tukey post‐hoc test: Distance: F(5, 25) = 146.3, *p* < 0.0001; Electrode: F(1, 5) = 7.460, *p* = 0.041; Interaction: F(5, 25) = 11.33, *p* < 0.0001), (C) Microglia protein ionized calcium‐binding adapter molecule 1 (Iba1) (Distance: F(5, 25) = 10.90, *p* < 0.0001; Electrode: F(1, 5) = 13.36, p = 0.015; Interaction: F(5, 25) = 12.16, p < 0.0001) and (D) Neuronal marker Fox‐3/ Rbfox3/ Hexaribonucleotide Binding Protein‐3 (NeuN) as a function of the distance from the electrode implantation site (Distance: F(5, 25) = 182.8, *p* < 0.0001; Electrode: F(1, 5) = 8.091, *p* = 0.036; Interaction: F(5, 25) = 5.731, *p* = 0.0012). (E) Mean distance to the nearest NeuN+ (mature neuron) nucleus from the electrode hole for each group (unpaired T‐test: t(10) = 3.354, *p* = 0.0073). Data are represented as mean ± SEM (*n* = 6). ^*^ *p* < 0.05, ^**^ *p* < 0.01, ^***^ *p* < 0.001, ^****^ *p* < 0.0001.

Histology revealed reduced encapsulation tissue surrounding the PEDOT:PTS coated electrodes with increased presence of peri‐electrode neurons. The density of astrocytes and microglia was significantly lower within a 250 µm radius of the edge of the coated electrode while neuron density within the region was significantly increased when compared with the uncoated electrode (Figure 5), consistent with improved biocompatibility of the coated electrode. The density of astrocytes, identified by GFAP, was significantly reduced surrounding the PEDOT:PTS coated electrode in comparison to PtIr electrodes (two‐way repeated‐measures ANOVA with Tukey post‐hoc test: Distance: F(5, 25) = 146.3, *p* < 0.0001; Electrode: F(1, 5) = 7.460, *p* = 0.041; Interaction: F(5, 25) = 11.33, *p* < 0.0001; Figure 5A,B). Similarly, when compared to the PtIr control, the PEDOT:PTS coated electrodes showed a reduction in microglia as identified through Iba1 staining (two‐way repeated‐measures ANOVA with Tukey post‐hoc test: Distance: F(5, 25) = 10.90, *p* < 0.0001; Electrode: F(1, 5) = 13.36, *p* = 0.015; Interaction: F(5, 25) = 12.16, *p* < 0.0001; Figure 5A,C). PEDOT:PTS electrodes also exhibited a significantly higher density of neurons around the area of the implant when compared with the PtIr electrode (two‐way repeated‐measures ANOVA with Tukey post‐hoc test: Distance: F(5, 25) = 182.8, *p* < 0.0001; Electrode: F(1, 5) = 8.091, *p* = 0.036; Interaction: F(5, 25) = 5.731, *p* = 0.0012; Figure 5A,D). The mean distance to the nearest mature neuron nucleus was significantly lower for coated electrodes than for uncoated electrodes (unpaired T‐test: t(10) = 3.354, *p* = 0.0073; Figure 5E).

An equivalent comparison between pristine PtIr and stimulated PtIr electrodes based on Evers et al., [21]. is presented in Figure S1. The GFAP stained area was significantly higher in the vicinity of stimulated PtIr electrodes, while neither the Iba1 stained area, the number of NSE positive nuclei nor the closest NSE positive nucleus were different between the two conditions.

#### 2.2.2 Effect of Encapsulation Tissue Properties and Neuron Distribution on Signal Amplitude

To examine separately the effects of the experimentally observed differences in encapsulation tissue and neuron distribution, LFP signals were simulated under three different conditions. In the first condition, neurons were integrated within the encapsulation tissue surrounding the electrode, in the second condition, neuronal cell loss was assumed to occur within the encapsulation tissue, and in the third condition, assumed to be the most representative of physiological conditions based on the available histological evidence (Figure 5 and [9, 21, 29]), neuron density was varied inversely proportional to the intensity of astrocytes and microglia within the encapsulation tissue, and a neuronal ‘kill zone’ where no neuronal sources were located was defined immediately surrounding the electrode, [26, 30].

**FIGURE 6:**
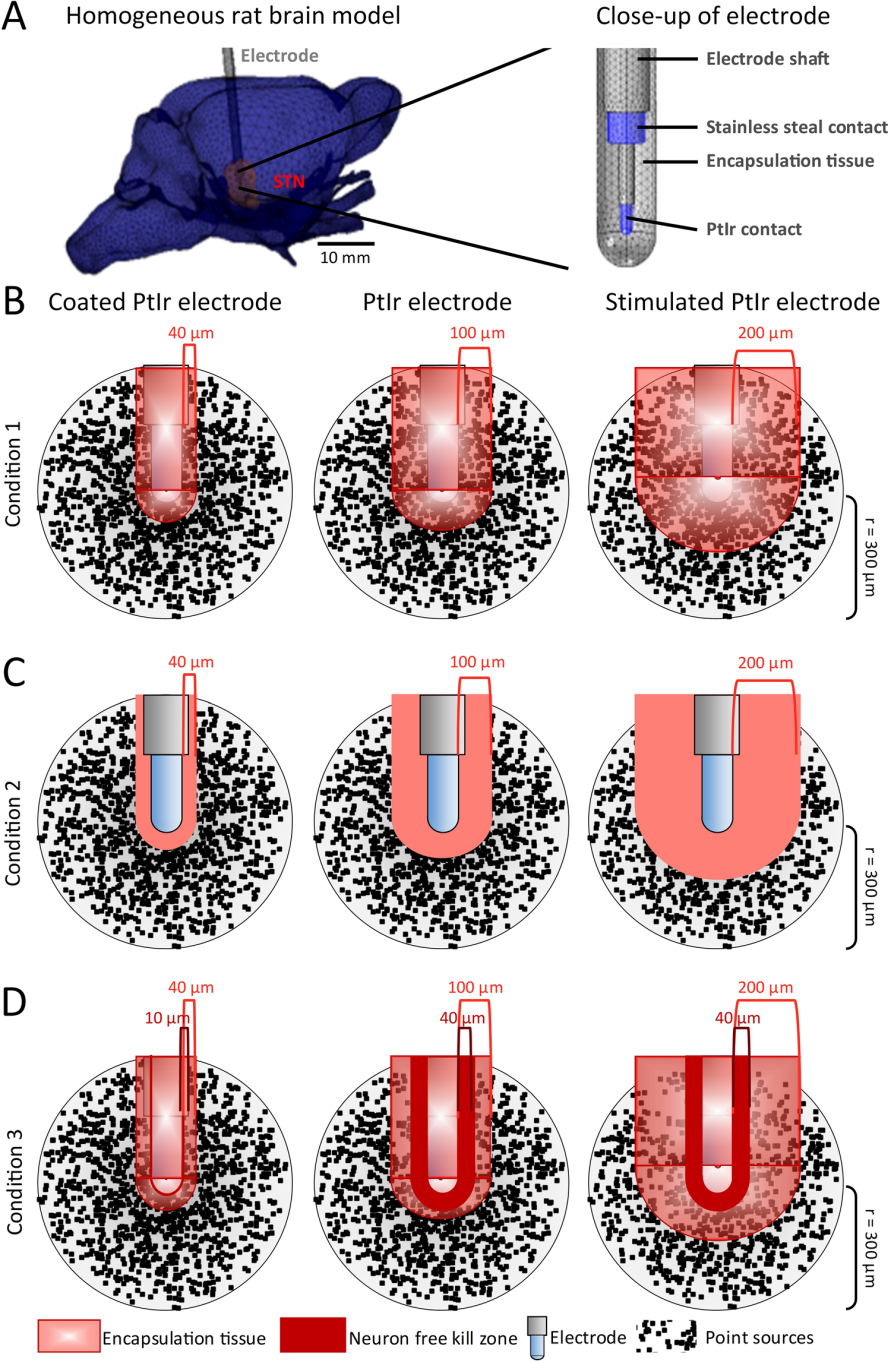
Simulation of the effect of encapsulation tissue properties and neuron distribution. (A) Finite element (FE) model model of the rat brain. Geometry of the rat brain model with a close‐up image of the implanted electrode surrounded by encapsulation tissue. Local field potential (LFP) signals were simulated for each electrode under three conditions. (B) Condition 1: Neurons located within the encapsulation tissue. The neural sources were randomly distributed within a sphere of 300 µm radius symmetrically centred on the tip of the electrode. (C) Condition 2: Neuronal cell loss within the encapsulation tissue. Neuronal sources were randomly distributed between the outer border of the encapsulation tissue and a 300 µm radius sphere centred on the electrode. (D) Condition 3: Variable neural density within the encapsulation tissue (transparent red). Neural sources were randomly distributed between the outer border of the ‘kill zone’ (dark red) [30] and the outer surface of a 300 µm radius sphere centred on the electrode. The density of the neuronal sources was reduced to 60% within the encapsulation tissue for the PtIr electrodes. For the PEDOT:PTS electrode a neural kill zone of 10 µm and a neuron density of 3000 points/mm^3^ were assumed. The corresponding values for the PtIr electrodes were 40 µm and 1800 points/mm^3^. The encapsulation tissue thickness was 40 µm for the PEDOT:PTS electrode, 100 µm for the PtIr electrode and 200 µm for the stimulated PtIr electrode for all conditions.

Through examination of the histological results presented in Figure 5, histological evidence from our previous work comparing the glial response of stimulated and unstimulated electrodes [21] and data on PEDOT coated electrodes from the literature [9, 21, 29], encapsulation tissue properties representing the glial structure and neural distribution around the chronically implanted electrodes were identified. We have previously shown that gliosis surrounding stimulated PtIr electrodes was denser and thicker after 8 weeks of continuous high‐frequency stimulation when compared to the encapsulation tissue surrounding unstimulated PtIr electrodes, with estimated encapsulation tissue thicknesses of 200 and 100 µm, respectively (Figure S1, also [21]). Neuronal loss was minimal and limited to a region of 100 µm from the electrode. The encapsulation tissue thickness was thus assumed to be 40 µm for the PEDOT:PTS coated electrodes (Figure 5), and 100 and 200 µm for the unstimulated and stimulated PrIr electrodes respectively for all three simulated conditions [21] Figure 6.

**FIGURE 7:**
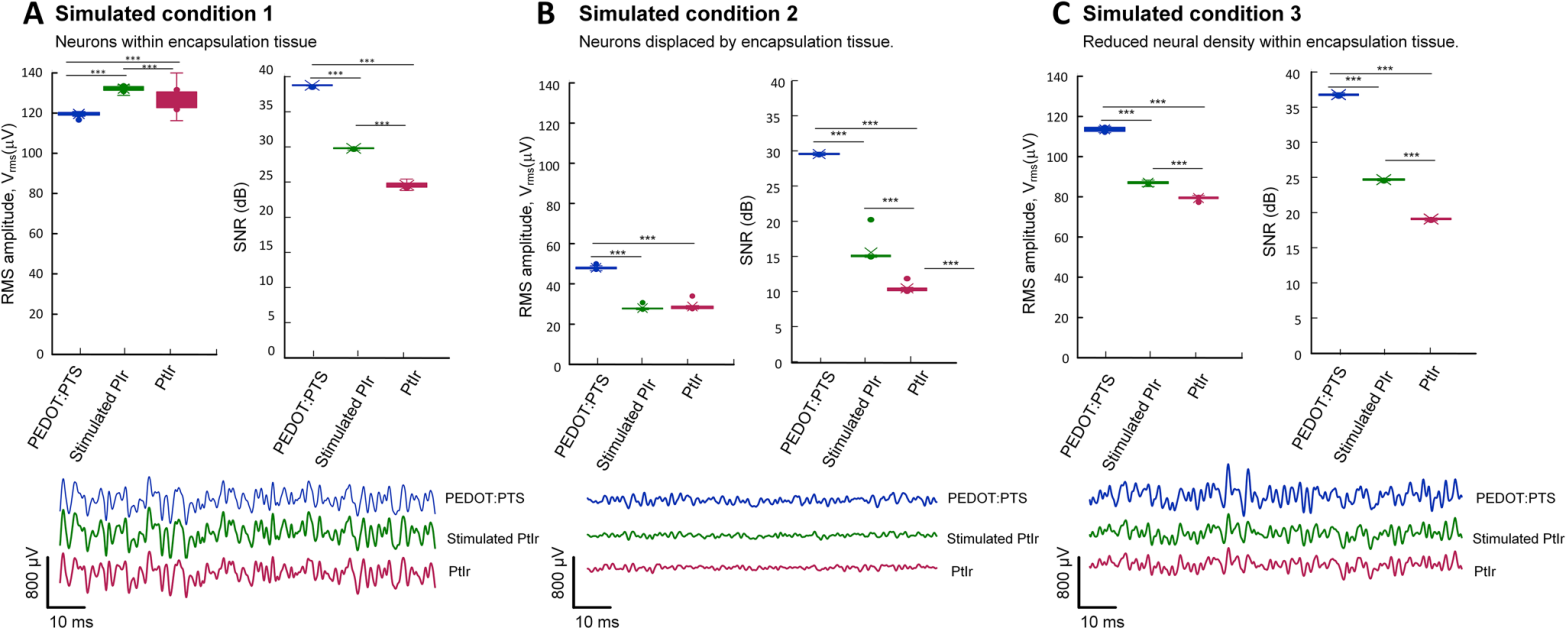
Simulated local field potential (LFP) root mean square (RMS) amplitude, signal‐to‐noise ratio (SNR) and simulated LFP for PEDOT:PTS coated, PtIr and stimulated PtIr electrodes. (A) Condition 1: Encapsulation tissue thickness varied with electrode type and neurons were present within the encapsulation tissue (one‐way ANOVA with Tukey post‐hoc test for comparisons V_rms_: F(2,27) = 711.8, *p* < 0.0001, SNR: F(2,27) = 980.8, *p* < 0.0001). (B) Condition 2: Encapsulation tissue thickness varied with electrode type based on histology. Neuronal cell loss was assumed within the encapsulation tissue and neurons were located within the grey matter bulk tissue only (V_rms_: F(2,27) = 711.8, *p* < 0.0001, SNR: F(2,27) = 980.8, *p* < 0.0001). (C) Condition 3: Encapsulation tissue varied with electrode type and neuron density within the encapsulation tissue was reduced (V_rms_: F(2,27) = 3441, *p* < 0.0001, SNR: F(2,27) = 50790, *p* < 0.0001). ^***^ *p* < 0.001. The results from 10 simulations with randomly distributed neuronal sources are presented.

Condition 1: Neurons embedded within the encapsulation tissue. When neurons were embedded within the low conductivity encapsulation tissue the amplitude of the simulated signal increased with the thickness of the encapsulation tissue and was highest for the PtIr electrode and lowest for the PEDOT:PTS electrode which had the thinnest encapsulation tissue Figure 7A. The mean value of the LFP RMS amplitude of the PEDOT:PTS coated electrode was 117.38 ± 1.18 µV with an estimated SNR of 37.31 ± 0.10 dB. The PtIr electrode exhibited slightly higher LFP RMS amplitude of 124.15 ± 6.16 µV, with a lower SNR of 23.04 ± 0.43 dB due to the higher thermal noise associated with the higher electrode impedance. The stimulated PtIr electrode, with the thickest encapsulation tissue, exhibited the highest LFP RMS amplitude of 130.05 ± 1.43 µV while the SNR of 28.32 ± 0.11 dB was lower than that of the PEDOT:PTS electrode but slightly higher than for the unstimulated electrode (one‐way ANOVA V_rms_: F(2,27) = 26.23, *p* < 0.0001, SNR: F(2,27) = 6829, *p* < 0.0001). The results reflect the influence of the conductivity of the tissue surrounding the sources on signal amplitude, with signal amplitude increasing with reduced tissue conductivity, and the combined effect of conductivity and thermal noise on the SNR.

Condition 2: Neurons located outside the encapsulation tissue. In the second condition, it was assumed that no active neurons were located within the encapsulation tissue. The distance between the closest active neurons and the electrode, therefore, varied with electrode type. For the PEDOT:PTS coated electrode, the mean RMS amplitude and SNR were 48.32 ± 0.89 µV and 29.57 ± 0.13 dB, respectively. Both RMS amplitude and SNR were lower for the PtIr electrode, 29.16 ± 1.79 µV and 10.44 ± 0.50 dB, respectively. Finally, though there was little difference in the amplitude of the simulated LFP signals for the stimulated PtIr electrodes, 28.37 ± 0.92 µV, thermal noise was lower, and hence SNR higher, due to the lower impedance of the stimulated electrode with respect to the unstimulated one, 15.53 ± 1.56 dB (one‐way ANOVA V_rms_: F(2,27) = 711.8, *p* < 0.0001, SNR: F(2,27) = 980.8, *p* < 0.0001). The higher signal amplitude observed for the coated PtIr electrode was due to a greater number of neurons located in close proximity to the electrode due to its thinner encapsulation tissue when compared with both of the uncoated PtIr electrodes Figure 7B.

Condition 3: Variation in neuron density depending on electrode biocompatibility. While conditions 1 and 2 enabled the effects of tissue electrical properties and distance between source and electrode to be examined in isolation, the histological data indicate that both neuron density and tissue properties varied in the immediate vicinity of the electrode. Therefore, a kill zone devoid of neurons was simulated for all electrodes along with reduced neural density in the encapsulation tissue, Figure 6D [30, 31]. The size of the area devoid of neurons was based on reports in the literature, which vary from no apparent kill zone to 100 µm, and histological data presented in Figure 5. Edell et al., studying 40 µm electrodes, observed a 10 µm kill zone for some implants, and 20–60 µm for others, after 6 months implantation in rabbits [32], while Stensaas and Stensaas [33] reported no neuronal loss for 500–750 µm diameter platinum electrodes implanted for 50–723 days in rabbits. Silicone probes implanted for 6 weeks in rats were surrounded by a 50–100 µm kill zone [34]. Wellman et al., 2019 reported reduced neural density < 100 µm for microelectrodes implanted in mice for 28 days [29]. Here we selected a kill zone of 10 µm for the coated electrodes and 40 µm for the PtIr electrodes in line with the minimal distance to the first neuron marked by NeuN (Figure 5E). The region was assumed to be the same for the unstimulated and stimulated PtIr electrodes as no differences in neuron distribution was observed previously [21]. PEDOT coatings have also been reported to reduce neuronal loss close to coated electrodes compared to uncoated electrodes. In vitro cell density on uncoated electrodes was reduced by 10%–15% [9]. Neuronal cell death was reduced by 37% around coated electrodes in the dorsal root ganglia [26]. In this study, a reduction in neuron density by 50%–66% for uncoated compared to coated electrodes was observed Figure 5C. Based on this the value of the density of the neuronal sources was reduced to 60% for the PtIr electrodes.

When the observed differences in electrode impedance and biocompatibility (encapsulation tissue thickness and neural distribution) were incorporated in the model, the signals simulated for the PEDOT:PTS coated electrode had the highest amplitude and SNR compared to the uncoated PtIr and stimulated PtIr electrodes. The LFP RMS amplitude for the PEDOT:PTS coated electrode was 113.42 ± 1.04 µV with a SNR of 36.99 ± 0.13 dB Figure 7C. In comparison, the RMS amplitude and SNR of the LFP signal from the PtIr electrode, with greater distance between active neurons and electrode interface, were 78.86 ± 0.87 µV and 19.07 ± 0.13 dB, respectively. Finally, for the stimulated PtIr electrode, with thicker encapsulation tissue and lower electrode impedance, the RMS amplitude and SNR of the LFP signals were 86.50 ± 0.86 µV and 24.74 ± 0.10 dB, respectively Figure 7C (one‐way ANOVA V_rms_: F(2,27) = 3441, *p* < 0.0001, SNR: F(2,27) = 50790, *p* < 0.0001).

#### 2.2.3 In Vivo Recorded LFP Signal Amplitude and Signal‐to‐noise Ratio

**FIGURE 8:**
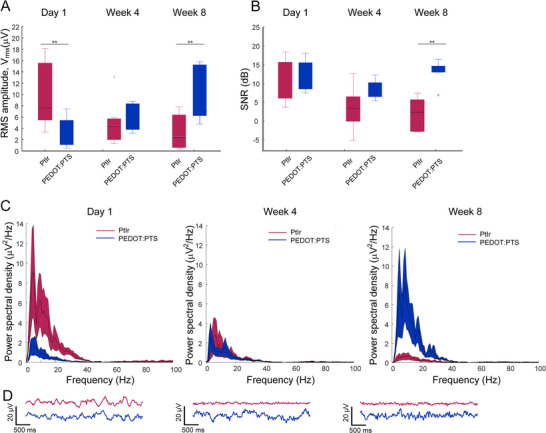
Local field potential (LFP) data recorded in vivo using chronically implanted PEDOT:PTS coated (*N* = 6) and uncoated PtIr electrodes (N = 7). (A) Amplitude of the recorded LFP at baseline (Day 1), 4 weeks and 8 weeks. A significant crossover interaction was observed (two‐way repeated‐measures ANOVA and Bonferroni post‐test, Coating: F(1,22) = 0.018, *p* = 0.90, Time: F(2,22) = 0.63, *p* = 0.54, Interaction: F(2,22) = 7.38, *p* = 0.0035) and (B) SNR for each electrode at baseline, 4 weeks and 8 weeks. SNR was significantly increased for coated electrodes at 8 weeks (two‐way repeated‐measures ANOVA and Bonferroni post‐test, Coating: F(1,22) = 8.63, *p* = 0.014, Time: F(2,22) = 9.36, *p* = 0.001, Interaction: F(2,22) = 6.85, *p* = 0.005). (C) Representative LFP signals and corresponding power spectra illustrating elevated LFP amplitude of the PEDOT:PTS coated electrode compared to PtIr electrode. Power line interference at 50 Hz and higher harmonics have been removed. Box and whiskers plots, whisker plots indicate 25–75 percentiles. (D) Representative examples of in vivo LFP recordings from the rat subthalamic nucleus at Day 1, week 4 and week 8.

Finally, LFP data recorded from PEDOT:PTS coated and PtIr electrodes chronically implanted in the rat basal ganglia were examined at three time points, at baseline immediately following electrode insertion, after 4 weeks and after 8 weeks of implantation Figure 8. The RMS amplitude of the LFP signal decreased over time for uncoated electrodes and increased for coated electrodes. This was evident by a significant crossover interaction on the two‐way repeated‐measures ANOVA (two‐way repeated‐measures ANOVA and Bonferroni post‐test, Coating: F(1,22) = 0.018, *p* = 0.90, Time: F(2,22) = 0.63, *p* = 0.54, Interaction: F(2,22) = 7.38, *p* = 0.0035 Figure 8A). Post‐hoc analysis revealed that the amplitude of the LFP signal detected with the coated electrodes was lower at baseline, similar at 4 weeks and higher at 8 weeks compared to the uncoated electrodes.

Improved performance of the coated electrode indicated by a significantly higher SNR was observed at the 8‐week time point (two‐way repeated‐measures ANOVA and Bonferroni post‐test, Coating: F(1,22) = 8.63, *p* = 0.014, Time: F(2,22) = 9.36, *p* = 0.001, Interaction: F(2,22) = 6.85, *p* = 0.005 Figure 8B). A higher SNR was observed for the coated electrode for which a higher number of neurons were present close to the electrode and less astrocytosis was observed, while a lower SNR was observed for the PtIr electrodes where stronger astrocytosis and loss of neurons were detected (Figure 5). The effect of electrode type observed experimentally correlated with simulated conditions 2 and 3, where higher signal amplitude and SNR were observed for the coated electrode due to the closer proximity of neurons to the conductive polymer coated electrode and lower thermal noise.

## 3 Discussion

We have used computational modelling in combination with in vivo and in vitro impedance, histology and electrophysiology data, to examine the different factors that affect the quality of neural signals recorded with conductive polymer coated electrodes. Using a computational model, the individual effects of electrode impedance, encapsulation and neuron distribution were examined in isolation, in a manner not possible using experimental methods alone. Though electrode impedance is commonly cited as the reason for poor signal quality, the results demonstrate that impedance alone does not influence the amplitude of the signal detected from neural current sources, provided that the input impedance of the recording amplifier is sufficiently high. Higher electrode impedance will, however, result in higher thermal noise, contributing to reduced SNR. Conductive polymer coatings are also associated with enhanced biocompatibility and better integration of the electrode into the surrounding tissue. Higher signal amplitudes were thus observed with the polymer coated electrodes when differences in neural distributions observed in the histological data were incorporated in the model simulations, with neurons present in closer proximity to coated than uncoated electrodes. The high resistivity of the encapsulation tissue further amplified the contribution of any neurons located within the encapsulation tissue. The simulation results agreed with experimental observations of higher signal amplitude and SNR at 8 weeks for polymer coated electrodes chronically implanted in the rat STN.

### 3.1 Impedance and Electrical Double Layer of Polymer Coated Electrodes

Polymer coating reduced electrode impedance without compromising the geometric dimensions of the electrode. The estimated value of parameter *K* in the constant phase element model which represents the double layer of PEDOT:PTS was found to be 70 times smaller than that of the uncoated PtIr electrode and 46 times smaller than the stimulated PtIr electrode. The substantial reduction in impedance of polymer coated electrodes at low frequencies is mainly attributed to an increased effective surface area, which, in turn increases the capacitance of the double layer [35, 36, 37]. The reduced impedance of the electrode under stimulation, on the other hand, can be attributed to protein desorption under electrical stimulation [21]. Incorporating the estimated double layer properties from the in vitro studies into the computational model yielded an overall electrode impedance that matched well with experimentally recorded impedances at 8 weeks Figure 1B.

The reduced impedance of the PEDOT:PTS electrodes resulted in substantially lower thermal noise for the polymer coated electrodes, with values of 1.67 and 8.7 µV estimated for the PEDOT:PTS coated and PtIr electrodes, respectively. The values were slightly lower than the range of 2.1–2.8 µV estimated by Neto et al., [11]. for PEDOT coated microelectrodes, which would be expected due to the lower surface area of 177 µm^2^ for the electrodes used in that study. The RMS value of the thermal noise of the PtIr electrodes following stimulation, 4.9 µV, was also lower than that of the unstimulated PtIr due to the associated reduction in EDL impedance [21].

### 3.2 Effect of Amplifier Impedance

Voltage division across the electrode interface and amplifier can lead to attenuation of the recorded signal if the amplifier input impedance is insufficiently high relative to the impedance of the electrode‐tissue interface [38]. Consistent with this, the model demonstrated a reduction in signal amplitude when the amplifier impedance was less than approximately ten times that of the electrode impedance. For the microelectrodes examined, a reduction in signal amplitude would be expected for amplifier impedances less than approximately 10 MΩ in the case of the unstimulated PtIr electrode and approximately 0.5 MΩ for the PEDOT:PTS coated electrodes Figure 2. In the experimental studies, and subsequent simulations, the 100 MΩ  input impedance of the amplifier was sufficiently high to avoid signal attenuation due to voltage division. It is possible that variations in the electrode interface‐amplifier impedance ratio may contribute to contradictory findings observed in experimental studies where both an effect of electrode impedance on signal amplitude [12] and no effect of electrode impedance [11] have been reported. In the development of fully implantable systems, the high impedance of very small microelectrodes combined with the technical constraints imposed by device miniaturisation can pose a challenge. For very small microelectrodes, the impedance at the electrode may indeed dominate such that voltage division occurs. The accompanying increase in thermal noise may result in a substantial reduction in SNR and signal quality. This can be mitigated in‐part by reducing electrode impedance through the application of conductive polymer coatings or similar treatments to reduce the amplifier input impedances required to avoid signal attenuation.

### 3.3 Effect of Electrode Impedance on Neural Recordings with Polymer Coated Electrodes

Understanding the potential influence of EDL properties on neural recordings is critical in the development of the next generation of brain machine interfaces and neuromodulation devices. Due to their low bandwidth and stability, LFPs can be used for brain computer interfaces to control assistive devices [39]. LFP‐derived biomarkers have also been examined in computational [40, 41, 42], preclinical [43] and clinical [44, 45, 46] studies to identify targets and establish closed‐loop or adaptive control of deep brain stimulation systems. While it is well‐established that the detection radius of recording electrodes is highly sensitive to the interelectrode distance and properties of the surrounding tissue [47, 48], the potential effects of electrode impedance and conductive polymer coating are less well understood. The spatial reach of the electrode was first investigated for a bipolar electrode configuration with both electrode contacts located in grey matter and neurons randomly distributed within a sphere centred mid‐way between the electrode contacts. For all three electrodes simulated, the maximum value of LFP RMS amplitude saturated when the radius of the active sources was approximately 1400 µm. Further increasing the radius containing active sources had little influence on the amplitude of the detected signal. Simulations were also conducted to represent an anatomical situation where recordings were performed within a contained region, such as the STN, with a single unipolar contact located in grey matter. In this case, the sphere containing active neural sources was centred on the distal contact and neurons were not located in the vicinity of the proximal contact. This yielded a more focal electrode detection volume of approximately 400 µm. In both cases, the electrode impedance had no discernible effect on either the amplitude of the detected signal or the detection volume of the electrode Figure 3C,D. The absence of a direct effect of electrode impedance on the amplitude of the detected signal was further confirmed in an inhomogeneous model of the rat brain which incorporated grey and white matter properties, and current sources with distributed weightings located in an anatomically‐based STN geometry Figure 4. Similar to what was observed here, Malaga et al., reported that increasing EDL resistivity by a factor of six resulted in only a 0.1% decrease in signal amplitude for simulated Utah arrays [17] while Lempka and McIntyre also observed that switching between a low and high impedance for a 100 µm‐thick interface layer had no effect on simulated LFP signals detected using larger DBS electrodes [48]. For this reason, the double layer at the interface of recording electrodes is typically neglected [49], though EDL impedance changes can have a substantial impact on the SNR through the effect on thermal noise.

### 3.4 In Vivo Electrode Impedance, Signal Quality and Immunohistology

Conductive polymers, including PEDOT:PTS, are biocompatible in nature and promote neuronal growth while reducing glial activation following chronic implantation [8]. The in vivo study presented here is unique in reporting three outcome measures, electrode impedance, signal quality and immunohistology, in the same animal. While short and long‐term effects of PEDOT coated electrodes have been reported in literature, most studies have included only one or two of these outcome measures [5, 7, 24, 50, 51, 52, 53]. The individual results presented show a reduction in impedance Figure 2, reduced foreign body response and increased presence of neurons in the electrode vicinity (Figure 4, and an increase in recording quality of the polymer coated electrode Figure 8.

The electrode impedance recorded in vivo in this study confirmed the electrochemical stability of PEDOT:PTS over a period of at least 8 weeks. Similar low and stable impedance has been observed for PEDOT coated microwire arrays with 25 µm diameter contacts implanted in the rat cortex by Venkatraman et al., [5]. over 12 weeks, where the impedance at 1 kHz remained constant, and for PEDOT coated silicon microelectrodes with 18 µm contacts by Kozai et al., [51]. over 4 months. A comparison of different coating materials found that PEDOT:PTS coated electrodes had the lowest impedance and highest SNR in an acute rat model of acoustic stimulation [24]. An additional study showed lower impedance and increased number of recorded units for PEDOT:TFB coated cortical electrodes over 12 weeks [7]. Zhou et al., studied multiwall carbon nanotube‐doped PEDOT coated microelectrodes and found a reduction in astrocyte intensity on histology by one third and a doubling in intensity of stained neurons [10]. The effect was slightly stronger than observed here, which may be explained by differences in electrode size and flexibility. PEDOT:PSS electrodes implanted in the cortex of rats had good recording quality for 21 days and histological examination showed a transitory microglia activation and limited progressive astrocytosis which remained reduced but no comparison to uncoated electrodes was made [50]. Forcelli et al., also report lower levels of astrocytosis around electrodes coated with poly(3,4‐ethylenedioxypyrrole) (PEDOP) at 14 days post implantation [53].

Conductive polymer coatings can degrade due to delamination or electrochemical stressors over time [4]. For potential clinical application longevity of the implant is paramount. To date, PEDOT:PTS has been shown to be electrochemically stable for 4 months using cyclic voltammetry and impedance spectroscopy in vitro [54]. In vivo, stable impedance and recording quality has been shown for PEDOT/CNT/Dex in rats over 15 months with no signs of delamination on electron microscopy after explant [55], stable impedance of PEDOT/TFB has been shown over 12 weeks in rats [7], PEDOT:PSS and PEDOT:CNT have been reported to retain stable impedance for 4 months in mice [51] and reliable electrophysiological signals have been recorded with several PEDOT:PSS based coatings in rats for 8 weeks [56]. For this study, no delaminated PEDOT material was observed on histological images, and the electrode coating was intact microscopically after electrode explant.

### 3.5 Influence of Encapsulation Tissue Properties and Neuronal Distribution on Recorded Signals

While the simulations described above allowed the effect of electrode impedance on the recorded signal to be examined in isolation from other factors, the histological data provide strong evidence for differences in both glial tissue properties and neuron distribution in the region surrounding polymer coated and uncoated PtIr electrodes. The glial scar formed around implanted devices can damage or displace nearby neurons, reducing the quality and SNR of the recorded signals [17, 57]. The histological results presented indicate that neurons remain closer to the PEDOT:PTS coated electrode and the gliosis surrounding PEDOT:PTS coated electrode is thinner compared to PtIr electrodes Figure 5.

To examine the combined effect of these different factors, the influence of encapsulation tissue with integrated neurons (condition 1), neuronal cell loss (condition 2), and reduced neuron density (condition 3) on the detected LFP signal was examined using the model, with electrode impedance and encapsulation tissue thickness matched to electrode type in each condition. When neuron sources were located within the encapsulation tissue (condition 1), the PEDOT:PTS electrodes exhibited lower amplitude LFP signals when compared to the other electrode conditions simulated Figure 7A. In this case, the amplitude of the detected signal increased with increasing thickness of the encapsulation tissue due to the higher resistivity of the encapsulation tissue. In contrast, with neuronal cell loss (condition 2) or reduced neural density close to the electrode (condition 3), the RMS amplitude of the simulated LFP signals of PEDOT:PTS electrodes was higher when compared with the PtIr electrodes due to reduced encapsulation tissue thickness and increased neuronal density Figure 7B,C. In each of the above simulated conditions, the SNR of the PEDOT:PTS electrode was superior to that of the PtIr electrodes Figure 7. The results indicate that the increased number of neurons in the vicinity of the electrode in combination with reduced glial encapsulation led to the recorded higher SNR of the PEDOT:PTS coated electrode. The neuronal loss and insulation by glial encapsulation tissue observed around the PtIr electrode, on the other hand, contribute to lower SNR.

Under simulated condition 3, PEDOT:PTS coated electrodes showed an increase in LFP amplitude and SNR compared to unstimulated and stimulated PtIr electrodes. The main reason for the elevated LFP amplitude was the increased density of neurons close to the electrode. Since this condition incorporated both loss of neuronal structures and neurons integrated within the encapsulation tissue, the encapsulation tissue conductivity and thickness had minimal effect on the resulting LFP amplitude. This scenario is physiologically the most likely based on the available histological evidence Figure 5.

Consistent with the model predictions, the in vivo LFP recordings exhibited significantly higher LFP amplitude and SNR for the coated electrodes in comparison to the PtIr electrodes at 8‐weeks in line with conditions 2 and 3 Figure 8. The RMS amplitude of recorded LFPs from the uncoated PtIr electrode decreased over time possibly due to the foreign body response and neuron degeneration around the implanted electrode. In contrast, the amplitude of the coated electrode increased over time, potentially due to greater biocompatibility of the polymer coating and neural growth closer to the electrode. This interdependency is seen in the significant effect of the interaction between electrode impedance and time on coated and uncoated LFP signal amplitudes at baseline and at 8 weeks.

For the stimulated PtIr electrode, LFP amplitude was slightly lower than that of the unstimulated PtIr electrode due to the thicker encapsulation tissue, while SNR was higher due to lower thermal noise caused by the reduced impedance due to protein desorption under stimulation [21]. The surface area of the stimulated and unstimulated electrodes was assumed here to be constant. However, protein absorption at the electrode may introduce an additional effect by occluding the electrode reducing the effective surface area. Johnson et al., reported an increase in SNR when a bias DV voltage of 1.5 V was applied to microelectrodes, but attributed the increase in SNR to decreased resistance of reactive encapsulation tissue followed by drop in thermal noise [18]. In contrast to condition 2 where there is a loss of neurons, when neurons were integrated inside the encapsulation tissue (condition 1) the model predicted higher LFP signal amplitude for the PtIr electrodes, with the highest amplitude observed for the stimulated PtIr electrode which had the thickest encapsulation tissue. This condition, however, is unlikely due to the neuronal loss around implanted electrodes. The SNR of the PEDOT:PTS electrode remained superior to that of the PtIr and stimulated PtIr electrode for both conditions.

### 3.6 Model Limitations

A number of model limitations should be considered when interpreting the results. The FE model used in this study assumes isotropic and homogeneous electrical properties for both the encapsulation tissue and rat grey and white matter tissue. Furthermore, the electrical double layer has non‐uniform electrical properties and cannot be fully represented mathematically. The double layer model used does not account for ion diffusion and assumes the PEDOT coating to be fully integrated with the electrode surface, which may not always be the case depending on the coating technique employed and chronic condition of surrounding tissues [58]. Biocompatibility was represented only through encapsulation tissue thickness and neural distribution in the model, other factors were not considered. Delamination and structural changes such as cracking are identified modes of failure for polymer coatings. The electrodes were examined after explanation and no evidence of delamination or defects in the coating were observed. Nevertheless, delamination would further alter the electrode tissue interface and the electrical and geometrical properties of the volume conductor in its immediate vicinity. The encapsulation tissue incorporated in the FE model was assumed to be uniform whereas it is a complex and inhomogeneous structure comprised of astrocytes, microglia, and oligodendrocytes [59]. Neuron density was necessarily based on histological evidence from several animal studies using different electrode materials [8, 9, 21, 29]. Additionally, as the focus here is on the effects of electrode coating only thermal noise was considered, whereas other noise sources such as shunt noise, pink noise, and biological noise will also effect the SNR of the electrode [58]. The contribution from external noise sources may dominate over thermal noise, however, these noise sources are not directly related to the electrode interface properties and are not considered here. In line with previous studies which have shown that the LFP is dominated by synaptic activity of populations of neurons in the region surrounding the electrode [48], synaptic activity of neurons was simulated using points source and the contribution of action potentials to the LFP was assumed to be negligible. A more physiological representation could be achieved with multi‐compartment neuron models and synaptic inputs spatially distributed throughout the dendritic arbor. However, while this would yield a more physiological description of the source, the effect of the electrical and geometrical properties of the electrode and tissue examined on signal amplitude would be unchanged.

Finally, in this study we have focused on the effect of polymer coated electrodes on LFP signals recorded from populations of neurons. The introduction of electrode coatings may be of more benefit for single cell recordings, which can be more sensitive to variations in noise and biocompatibility associated with different electrode‐tissue interface properties. The amplitude of the recorded extracellular potential from a given source should, nevertheless, be similarly insensitive to the impedance of the electrode‐tissue interface for both single cell and population recordings [48].

## 4 Conclusion

Understanding the mechanisms underlying charge transfer at the electrode‐tissue interface is critical to improve the longevity of implantable brain machine interfaces. A greater understanding of how conductive polymer coatings influence neural signal properties has the potential to improve and optimize brain machine interfaces which is needed to facilitate more widespread adoption of the technology. In model simulations and experimental recordings of LFP signals, PEDOT:PTS coating of neural recording electrodes was shown to improve recording quality and electrode performance due to enhanced biocompatibility when compared with PtIr electrodes, which are associated with protein adsorption, peri‐electrode neurodegeneration and greater gliosis. While electrode impedance alone did not affect electrode detection volume or signal amplitude, for very small microelectrodes the use of coatings and subsequent reduction in impedance can help to avoid signal attenuation due to voltage division across the electrode and amplifier, while also reducing thermal noise. Overall, the results indicate that improved biocompatibility expressed as reduced gliosis and increased neuron density close to the implanted electrode play a larger role in improving signal quality, increasing signal amplitude and SNR, than reducing electrode impedance.

## 5 Methodology

**FIGURE 9:**
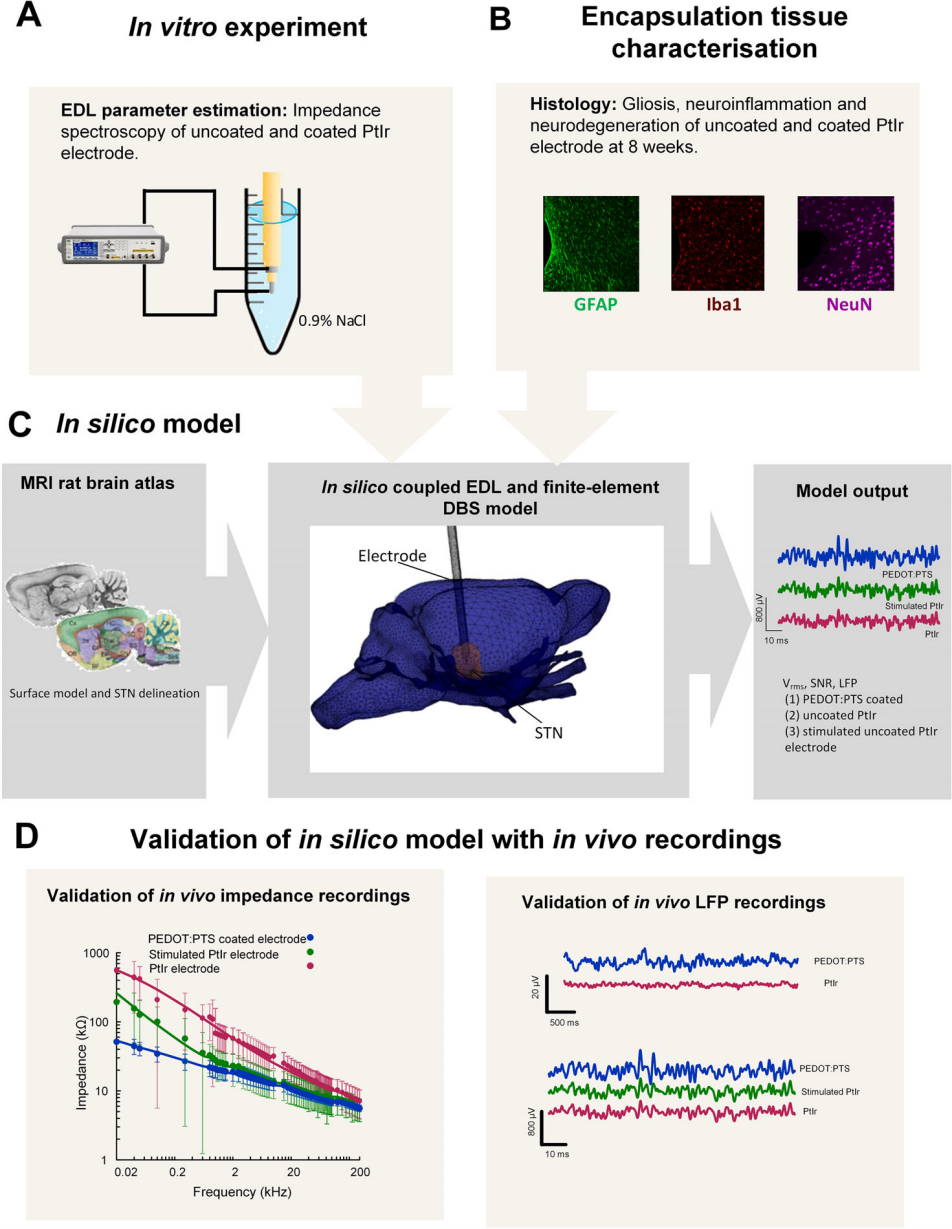
Integration of in vitro, in vivo, and in silico models. (A–C) The workflow shows the inputs and outputs of the in silico model. (D) In vivo data used to validate the in silico model.

LFP signals recorded using PEDOT:PTS coated, uncoated PtIr and stimulated uncoated PtIr electrodes located in the rat STN were simulated using a FE volume conductor model of the rat brain. The volume conductor model was coupled to synaptic currents, represented as point current sources randomly distributed in the region of tissue surrounding the electrode. The EDL properties of PEDOT:PTS coated PtIr electrodes were estimated from in vitro impedance data recorded from a coated electrode in 0.9% saline solution (*N* = 5). The EDL was incorporated into the FE model using the thin layer approximation [60], similar to the method describes in Evers et al., [21]. The EDL properties of the stimulated and unstimulated uncoated PtIr electrodes were adopted from Evers et al., 2022^22^. The thickness of the encapsulation tissue surrounding the electrode was estimated from the histology of the encapsulation tissue surrounding PEDOT:PTS coated (*N* = 6) and uncoated PtIr electrodes (*N* = 7) chronically implanted in the rat brain Figure 5. The integration of the in silico, in vitro and in vivo models is presented in Figure 9.

### 5.1 Experimental Methods

#### 5.1.1 In Vitro Characterization of Electrical Double Layer Impedance

The electrical double‐layer of 5 PEDOT:PTS coated bipolar PtIr electrodes (SNEX‐100, Microprobes for Life Science) was characterized in 0.9% saline solution at room temperature. The electrode tip Ø 100 µm with an active surface area of 0.078 mm^2^, was located 0.5 mm from a stainless‐steel reference contact with a surface area of 0.34 mm^2^. Electrode impedance spectra (20 Hz—300 kHz) were measured using a precision LCR Meter (E4980AL, Keysight) with an applied current amplitude of 20 µA in each electrode. Similarly recorded data from uncoated PtIr electrodes of the same type reported in Evers et al., [21]. were used for comparison.

The recorded impedance data were then incorporated in a FE model of the in vitro experimental setup to estimate the EDL properties of the electrode‐electrolyte interface. The FE model of the in vitro experimental setup comprised a concentric coated electrode located at a depth of 1 cm in a cylindrical domain representing a 10 cm long plastic test tube, 2.6 cm in diameter, filled with 0.9% saline solution with a conductivity of 1.45 S m^−1^. To estimate the equivalent EDL parameters, the impedance spectrum of the electrode in 0.9% saline solution was fit to the circuit model proposed by McAdams et al., [27].

(1) $$Z_{cpe}=K{\left(j\omega \right)}^{-\beta }$$

(2) $$R_{ct}=\frac{RT}{nFI_{o}}\;$$

where *Z_cpe_* is the constant phase angle element, *R_ct_* the charge transfer resistance, and *K* and β are constants denoting the magnitude of the impedance of the EDL and inhomogeneities at the surface of the electrode, respectively; *R* is the universal gas constant, *F* Faraday's constant, T temperature, *n* the number of electrons per molecule, and *I_o_* the exchange current density. The electrode was considered to be partially polarizable where the electrode behaves as a pseudo‐capacitor, and the faradic component was assumed to be zero due to electrode reaction being infinitely slow. The estimated EDL parameters for the coated and uncoated electrodes were then used in the heterogeneous FE rodent brain model described in section 5.2.

#### 5.1.2 In Vivo Experiments

Impedance spectroscopy, STN LFP and immunofluorescence data were compared between PEDOT:PTS coated electrodes and uncoated PtIr electrodes. Thirteen naïve adult male Wistar rats (weighing 385 g ± 55.5 g (mean ± SD) and 9 ± 2.5 weeks old at the beginning of the experiment, housed in a SPF facility). PEDOT:PTS coated electrodes were implanted in 6 rats and identical uncoated electrodes in seven rats. Immunofluorescence was performed at 8 weeks to examine neurons (with Fox‐3/ Rbfox3/ Hexaribonucleotide Binding Protein‐3 (NeuN)), astrocytes (with glial fibrillary acidic protein (GFAP)) and microglia (with allograft inflammatory factor 1 / ionized calcium‐binding adapter molecule 1 (AIF‐1/ Iba1)) in the vicinity of the implanted electrodes. Data from uncoated electrodes implanted in six rats at which a stimulation current (pulse amplitude 40–100 µA, duration 60 µs and frequency 130 Hz) was continuously applied were included for comparison [21]. A randomized block design was used. Treatment and measurement order varied daily. Blinding to electrode type was not performed. All animal experimentation was covered under Health Product Regulatory Authority of Ireland license (AE18982‐P122) and University College Dublin Animal Research Ethics Committee approval (AREC 17–22).

##### 5.1.2.1 Electrodeposition of PEDOT:PTS

Bipolar PtIr electrodes (SNEX‐100, Microprobes for Life Science, Gaithersburg, USA) were coated with PEDOT:PTS prepared from 0.05 M EDOT (Sigma Aldrich, Ireland) and 0.1 M PTS (Sigma Aldrich, Ireland, 70 000 g mol‐1 MW) in acetonitrile and water (50:50 vol%). Electrodeposition was performed in an in‐house electrochemical cell, connected to a Princeton Applied Research Potentiostat/Galvanostat model 2273 controlled with Power Suite software. The SNEX‐100 electrode was used as the working electrode and a platinum foil (Goodfellow) as reference electrode.

##### 5.1.2.2 Electrode Implantation

During one recovery surgery, electrodes were implanted in the STN as described by Evers et al., [21]. Briefly, under isoflurane inhalation anaesthesia, the skull points Bregma and Lambda were exposed in a stereotaxic frame to locate the STN (−3.6 mm anteroposterior and −2.5 mm mediolateral from Bregma and −7.6 mm dorsoventral). A PEDOT:PTS coated or pristine PtIr bipolar electrode was inserted into the STN. Four bone screws were inserted to hold a cap of dental cement covering the skull. Six rats were implanted with PEDOT:PTS coated PtIr electrodes, seven rats were implanted with uncoated PtIr electrodes that were not stimulated. Impedance and histological data from six rats implanted with uncoated PtIr electrode at which a current was applied at 130 Hz, 60 µs pulse duration, 7 h/day was included for comparison [21]. Impedance and LFP data were recorded up to 8 weeks post‐surgery.

#### 5.1.3 Impedance Spectroscopy

The electrode impedance spectrum from 20 Hz—300 kHz was recorded daily for 10 days and three times weekly thereafter using a precision LCR Meter (E4980AL, Keysight, Santa Rosa, USA) at a current amplitude of 20 µA.

#### 5.1.4 Local Field Potential Acquisition and Analysis

Bipolar LFPs were recorded at implantation, and at 4 weeks and 8 weeks post‐surgery under brief isoflurane anaesthesia. Data were amplified using a pre‐amplifier (NL844, Digitimer Ltd, Hertfordshire), then further amplified and low pass filtered at 1 kHz (Neurolog, Digitimer Ltd, Hertfordshire), before A‐D conversion using the 1401 data acquisition unit and Spike2 acquisition software (C.E.D.,Cambridge, UK). Data were recorded at a sampling rate of 2.5 kHz with a 50 Hz notch filter applied.

During post‐processing the LFP signals were bandpass filtered between 2 Hz and 500 Hz using a fourth order Butterworth filter, and a notch filter was applied at 50 Hz and its higher harmonics to remove power line interference. The RMS amplitude of the filtered LFP signals was calculated over a 5 s epoch and the power spectra of the raw LFP signals were estimated for the same segment of data using the Fast Fourier transform using Welch's method with overlapping Hanning windows, 1 s in duration with 50% overlap. As the noise and LFP signals cannot be recorded in isolation in vivo, the SNR of experimentally recorded LFP signals, *SNR_exp_*, was estimated as the ratio of the power in the frequency range 1–250 Hz which contained most of the LFP signal power, *P_signal_*, to the power in the frequency range 251–500 Hz which was assumed to be predominantly noise, *P_noise_*.

(3) $$SNR_{exp}=10log10\left(\frac{P_{signal}}{P_{Noise}}\right)$$

#### 5.1.5 Histological Basis for Estimation of Encapsulation Tissue Thickness and Composition

Encapsulation tissue thickness and composition were estimated for PEDOT:PTS coated PtIr electrodes (*N* = 6) and for uncoated PtIr electrodes (*N* = 6, one block excluded due to insufficient sample quality). Similar values estimated for stimulated uncoated electrodes, representing the condition which occurs when a recording electrode is also used for stimulation or the electrode designation changes over time for example in closed‐loop systems or during reprogramming, were also incorporated within the models using values adapted from Evers et al., [21].

#### 5.1.6 Fluorescent Immunohistochemistry

After 8 weeks rats were cardially perfused with formalin to harvest the brains for immunofluorescence. Paraffin‐embedded horizontal 10 µm thick brain slices through the electrode tract were stained. After heat mediated antigen retrieval in Tris/EDTA solution (10/1 mM; pH9), non‐specific target block with 3% of bovine serum albumine (BSA) and 0.1% tween‐20 in phosphate buffered saline (PBS‐T), primary antibodies specific to neurons (NeuN, abcam, ab177487, 1:1000), astrocytes (GFAP, Sigma, 68893, 1:500) or microglia (Iba1, Wako, 019–19741, 1:500) were incubated and visualized with their corresponding secondary antibodies (Alexa Fluor 488, Invitrogen, A‐21202, 1:500 and/or Alexa Fluor 594, Invitrogen, A‐21207, 1:500). Tissue sections were scanned with a fluorescence scanning microscope to facilitate quantitative analysis using ImageJ (W.RasBand, National Institute of Health, Bethesda, US). During analysis all distances are reported from the edge of the void created in the blocks when the electrodes were removed.

### 5.2 Computational Models of LFPs Recorded With PEDOT:PTS Coated and Uncoated PtIr Electrodes

A FE model of the rat brain with a concentric bipolar microelectrode located within the STN was developed to simulate LFPs detected at the electrode with and without PEDOT:PTS coating. A geometric model of the microelectrode (SNEX‐100 concentric bipolar electrode, Microprobes, Gaithersburg, USA), was constructed, composed of an active PtIr contact with a tip diameter of 100 µm and an active surface area of 0.078 mm^2^. A similar geometry was created for the coated electrode, with the PtIr contact coated with PEDOT:PTS of 10 nm thickness. Both electrodes used a stainless‐steel reference contact with a diameter of 330 µm, length 0.25 mm and surface area of 0.34 mm^2^. The active electrode contact and reference steel contact were separated by 0.5 mm of polyimide tubing with a diameter of 140 µm.

The electrodes were positioned within a rat brain model composed of homogeneous grey matter, based on the Waxholm Space atlas of the Sprague Dawley rat Brain (WSSD) [61]. The electrode was located within the STN and surrounded by 200, 100 or 40 µm thick encapsulation tissue representing the glial scar formed due to chronic implantation of a stimulated PtIr, unstimulated PtIr, and PEDOT:PTS coated electrode, respectively, as estimated from the histological data. Electrical ground was positioned at the medulla.

LFP signals from neurons located in the tissue surrounding the electrode were simulated for each of the electrode conditions. Each neuron was represented using a point source current representing the summation of synchronized synaptic currents of that neuron.

#### 5.2.1 Material Properties

The electrical properties of the rat brain grey matter and encapsulation tissue were incorporated in the FE model using the 4 pole Cole‐Cole model with parameters described by Gabriel et al., and Evers et al., [21, 62]. Models were constructed using both homogenous brain tissue, and heterogeneous brain tissue comprised of grey and white matter and cerebrospinal fluid. In the homogeneous model, the conductivity and relative permittivity of brain tissue were assumed to be 0.25 S/m and 1×10^5,65,^ respectively, based on in vivo impedance measurements in the rat brain. In the heterogeneous model, the conductivity and relative permittivity of grey and white matter were estimated using the parametric model presented by Gabriel et al., [63], at 160 Hz which corresponds approximately to the median frequency of the simulated and experimental STN LFP signals. The encapsulation tissue and double layer electrical properties in both models were also estimated at 160 Hz Table 2. The PtIr contact, stainless steel ground contact and Polyimide insulation were assumed to be purely conductive with an electrical conductivity of 4.5×10^6^, 5×10^4^, 9×10^5^ and 1×10^−6^ S m^−1^, respectively [63, 64]. The electrical and geometrical parameters of the homogeneous and heterogeneous finite element brain models are summarized in Table 2.

**TABLE 2:** Electrical and geometrical properties of the materials included in the finite element models. Conductivity, σ, and relative permittivity, ε_r_, values are presented for the homogenous and heterogeneous brain models. The model boundary conditions are also presented. ^†^Values estimated at 160 Hz [21, 62].

Category	Description	Value
Material Properties	Brain tissue (homogeneous model), σ [64]	0.25 S/m
	Gray matter, σ ^†64^	0.093 S/m
	White matter, σ ^†64^	0.06 S/m
	Cerebrospinal fluid, σ [65]	2.0 S/m
	Encapsulation tissue, σ ^†22^	0.03 S/m
	Brain tissue (homogeneous model), ε_r_ [64]	1 × 10^5^
	Gray matter, ε_r_^†64^	1.7 × 10^6^
	White matter, ε_r_^†64^	7.6 × 10^5^
	Cerebrospinal fluid, ε_r_ [66]	108
	Encapsulation tissue, ε_r_^†22^	2 × 10^6^
	Saline (0.9%), σ	1.4 S/m
	PtIr contact, σ [63, 64]	5 × 10^6^
	Steel contact	9 × 10^5^
	Polyimide [67]	1 × 10^−6^
Electrode EDL Properties of *Z_cpe_*	K (CPE magnitude constant), PEDOT:PTS	0.031 Ωm^2^s^−^ᵝ
	K (magnitude constant), PtIr (unstimulated)	2.14 Ωm^2^s^−^ᵝ
	K (magnitude constant), PtIr (stimulated)	1.42 Ωm^2^s^−^ᵝ
	β (phase exponent), PEDOT:PTS	0.60
	β (phase exponent), PtIr (unstimulated)	0.67
	β (phase exponent), PtIr (stimulated)	0.85
Electrode Geometric Dimensions	Electrode tip diameter	100 µm
	Active surface area	0.078 mm^2^
	Reference contact diameter	330 µm
	Reference contact length	0.25 mm
	Reference contact area	0.34 mm^2^
	Inter‐electrode spacing	0.5 mm
	Polyimide insulation outer diameter	140 µm
	PEDOT:PTS coating thickness	10 nm
	Encapsulation thickness, PEDOT:PTS	40 µm
	Encapsulation thickness, PtIr	100 µm
	Encapsulation thickness, Stimulated PtIr	200 µm
Boundary Conditions	Outer surface insulating boundary	*n*.*J* = 0
	Electrode–tissue interface (thin layer approximation) [60]	$-n.J=\frac{1}{Z_{cpe}}\;\left(\phi_{tissue}-\;\phi_{elec}\right)$
	Electrical ground (medulla)	ϕ = 0

#### 5.2.2 Governing Equations

The extracellular potential, ϕ, at each electrode was simulated using the electro‐quasistatic formulation of the Laplace equation, whereby magnetic, inductive and wave propagation effects generated by synaptic currents in extracellular space were assumed to be negligible at the frequencies of interest [66, 68, 69].

(4) $$\nabla \cdot \;\left[\left(\sigma +\omega \varepsilon_{0}\varepsilon_{r}\right)\nabla \phi \right]=0$$

σ and ε_*r*_ are electrical conductivity and relative permittivity, ω is angular frequency, ε_0_ permittivity of free space, and φ the scalar potential.

The electrical double layer at the interface of the tissue and the PEDOT:PTS and PtIr electrodes was incorporated in the model using the thin layer approximation [60] with the parameter values given in Table 1. The model of the double layer is based on an empirical model of pseudocapacitive impedance. It incorporates both the Helmholtz layer and the Gouy‐Chapman diffuse layer and has been shown to provide a good fit for a range of electrode materials.

#### 5.2.3 Source Excitation and Boundary Conditions

Local field potentials represent the summation of the extracellular potentials generated by synaptic and transmembrane currents of neurons in the vicinity of the recording electrode [70, 71, 72, 73]. The LFP is assumed to primarily reflect the weighted synaptic activity of populations of neurons, with the dominant component arising from correlated synaptic inputs [72]. For electrodes located sufficiently far from the source, the contribution of each neuron can thus be approximated using a stationary point current source [72]. In the model, the net excitatory and inhibitory synaptic activity of each neuron was represented as a normally distributed Gaussian signal sampled at 20 kHz and band‐pass filtered using a third order Butterworth filter with low and high frequency cut‐off frequencies of 1 and 300 Hz. The filtered current source was scaled to have a standard deviation of 1 nA.

#### 5.2.4 Finite Element Model and Source Excitation Coupling

Taking advantage of the linear nature of the problem and the principle of reciprocity [17, 48], the potential recorded at the electrode due to a point current source representing the synaptic activity of a given neuron was evaluated as the potential at that neuron due to a current source at the electrode. This allowed the electromagnetic forward problem to be solved simultaneously for an arbitrary number of sources just once per electrode. The bipolar LFP signal generated by a population of *N* neurons was then calculated as

(5) $$\phi_{\mathrm{LFP}}\left(t\right)=\sum_{i=1}^{N}\left(\phi_{1}^{i}-\phi_{2}^{i}\right){\;*I}_{\mathrm{syn}}^{i}\left(t\right)$$

where ϕ_LFP_(*t*) represents the simulated LFP, $\;\phi_{1}^{i}$ and $\phi_{2}^{i}$ represent the potential at the two electrodes due to a synaptic current source at neuron *i* of unit amplitude and $I_{syn}^{i}\;\left(t\right)$ represents the synthetically generated synaptic current for neuron *i*.

#### 5.2.5 Finite Element Mesh and Solver

The geometric model of the rat brain and microelectrode was discretized with 0.8 million tetrahedral elements using COMSOL Multiphysics (COMSOL, USA). The discretized FE model was solved using COMSOL Multiphysics using the MUMPS (Multifrontal Massively Parallel Sparse Direct Solver) direct solver with Newton method. The total computation time required to solve the homogeneous rat model was approximately 180 s. The total physical memory used was 2 GB and the processor used was Intel R Xeon R with 2 processors with speed of 2.7 GHz on Windows Server 2012. The scalar electric potential was estimated using quadratic interpolation on each tetrahedral element.

#### 5.2.6 Thermal Noise

The amplitude of Johnson‐Nyquist noise, commonly referred to as thermal noise, due to the thermal motion of electrons within the electrode material, tissue medium and electrode‐tissue interface [22] was estimated based on the measured electrode impedance across the frequency range of interest and added to the simulated LFP signal. The RMS amplitude of thermal noise, *v*_*t*(*RMS*)_, across the frequency band f1 to f2 was estimated as [22],

(6) $$v_{t\left(RMS\right)}=\sqrt{4k_{b}T\int_{f_{1}}^{f_{2}}Re\left(Z\right)df}\;$$

where *k_b_* is the Boltzmann constant, T is temperature, *Re*(*Z*) is the frequency‐dependent real component of the impedance of the electrode interface [74], and *f_1_* and *f_2_* correspond to the lower and upper limits of the frequency band. A Gaussian random noise signal with RMS amplitude equal to *v*_*t*(*RMS*)_ was added to each simulated LFP signal, with *v*_*t*(*RMS*)_ assumed to be proportional to the square root of the electrode impedance as described in Equation (6).

#### 5.2.7 Signal‐to‐Noise Ratio

The *SNR* of the simulated LFP signal was estimated as the ratio of the power of the simulated LFP signal to the power of the background electrical noise, represented here by the thermal noise.

(7) $$SNR=10log10\left(\frac{P_{signal}}{P_{Noise}}\right)$$

where *P_signal_* and *P*_Noise_ are the power of the filtered LFP signal and noise signal, respectively.

#### 5.2.8 Estimation of Spatial Reach of LFP Signals

To estimate the spatial reach of the detected LFP signals, the active neurons were assumed to be randomly located within a spherical volume centered midway between the proximal and distal electrode contacts. The radius of the sphere was progressively increased in steps of 100 µm while keeping the density of neural sources within the volume constant and the RMS amplitude of the resulting LFP signal, simulated for a homogeneous volume conductor with uniform weighting of current sources, was estimated Figure 3. The detection radius of the electrode was defined as the radius for which the LFP signal detected at the electrode was 95% of that detected when all sources within the maximum radius of 500 or 1500 µm were activated.

#### 5.2.9 Simulation of LFP Signals Under Conditions of Varying Encapsulation and Neural Distribution

To simulate the first condition, encapsulation tissue properties for each electrode type (coated, uncoated, uncoated and stimulated) were assigned based on the histological data for the coated and uncoated electrodes and neural sources were permitted within the encapsulation tissue as well as in the surrounding grey matter Figure 5. Encapsulation tissue properties for the stimulated and unstimulated PtIr electrodes, specifically the reduced conductivity of the encapsulation tissue in comparison to normal grey matter, were adapted from Evers et al., [15]. Encapsulation tissue thickness was assumed to be 40 µm around the PEDOT:PTS electrodes, 100 µm around the PtIr electrodes and 200 µm around the stimulated PtIr electrodes. The neural sources were randomly distributed around the electrode within a sphere of a radius of 300 µm centred on the most distal tip of the electrode, with a uniform density of 3000 sources/mm^3^ throughout the volume Figure 6B. The SNR of the LPF signal was estimated based on the root mean square (RMS) amplitude of the thermal noise as described in section 2.4.

In the second condition, neurons were assumed to be absent from the encapsulation tissue. The encapsulation tissue thickness was similarly assumed to be 40, 100, and 200 µm for the PEDOT:PTS, PtIr and stimulated PtIr electrodes, respectively. Neural sources with a constant density (3000 points/mm^3^) were located between the outer surface of the encapsulation tissue and a sphere of radius 300 µm centred on the most distal tip of the electrode Figure 6C.

In the third condition (Figure 6D), neurons were allowed within the encapsulation tissue. The biocompatible nature of PEDOT:PTS coated electrodes allows for neuronal growth while reducing glial activation following chronic implantation [20]. Based on immunohistology data on the properties of the encapsulation tissue and neural density surrounding the electrode from long‐term implantation of PEDOT:PTS coated electrodes (Figure 5) and literature [15, 21, 22], it was thus assumed that the PEDOT:PTS electrode had a higher density of neurons in the encapsulation tissue and for the PtIr electrodes the density was reduced by 60% compared with the PEDOT:PTS electrode (estimated from Figure 5C as 50%–66% reduced neurons around the uncoated electrodes). For the PtIr electrode the neuronal kill zone was assumed to be 40 µm. Under condition 3, the density of neurons within the encapsulation tissue varied depending on the electrode type. The encapsulation tissue thickness was assumed to be 40, 100, and 200 µm for the PEDOT:PTS, PtIr and stimulated PtIr electrodes as before; however, a higher neuron density in the encapsulation tissue of 3000 neuronal sources/mm^3^ was assumed for the PEDOT:PTS coated electrode, with 1800 neuronal sources/mm^3^ for the uncoated PtIr electrodes. Outside the encapsulation tissue the neuronal source density was 3000/mm^3^ for all electrodes as in condition 1. A kill zone, in which no neurons are present, was also defined for each electrode type and extended to 10 µm for the coated electrode and 40 µm for both unstimulated and stimulated PtIr electrodes (Figure 5D and [27]).

### 5.3 Statistical Analysis

The required sample size for experimental studies was determined as N = 6 a priori based on in vitro impedance performance of the coated and uncoated electrodes. For the normally distributed impedance data and LFP parameters, a two‐way repeated‐measures ANOVA was performed with the factors coating and time or coating and frequency followed by Bonferroni post‐hoc testing if indicated. Histological results were analysed using a two‐way repeated‐measures ANOVA with Tukey post hoc test. Group comparison of the mean minimal distance of neurons from the electrode was done in an unpaired T ‐test. For the histology, the experimental unit was the hemisphere in which the electrode was implanted. Data are represented as mean ± standard deviation (SD) but for histological results where the standard error of mean (SEM) is reported. Power was set at 1‐β = 80% and the criterion for statistical significance was set at *p* ≤ 0.05.

Simulations were run ten times and data reported as mean ± SD. Comparisons between simulation outcomes were performed using a two‐way ANOVA. MATLAB (MathWorks, Natwick, USA) and Prism 7 (GraphPad Software, La Jolla, USA) were used for statistical analysis.

## Funding

The work was funded by Research Ireland and the European Regional Development Fund (Grant Number 13/RC/2073) and the European Research Council (Grant Number ERC‐2014‐CoG‐646923).

## Conflicts of Interest

The authors declare no conflict of interest.

## Supporting information




**Supporting File**: adhm70932‐sup‐0001‐SuppMat.docx

## Data Availability

The data that support the findings of this study are available from the corresponding author upon reasonable request.
