# Supplementary material for: Understanding the Effects of Conductive Polymer Electrode Coating on Recorded Neural Signals
Source: Adv Healthc Mater. 2026 Feb 21;15(17):e03893. doi: 10.1002/adhm.202503893 (PMC13175291; doi:10.1002/adhm.202503893)
Supplement: Supplementary file 1 — Supporting File: adhm70932‐sup‐0001‐SuppMat.docx [file ADHM-15-0-s001.docx]

**Supplemental data**

Karthik Sridhar, Judith Evers, Alexandre Trotier, Manus Biggs, Madeleine M. Lowery

**
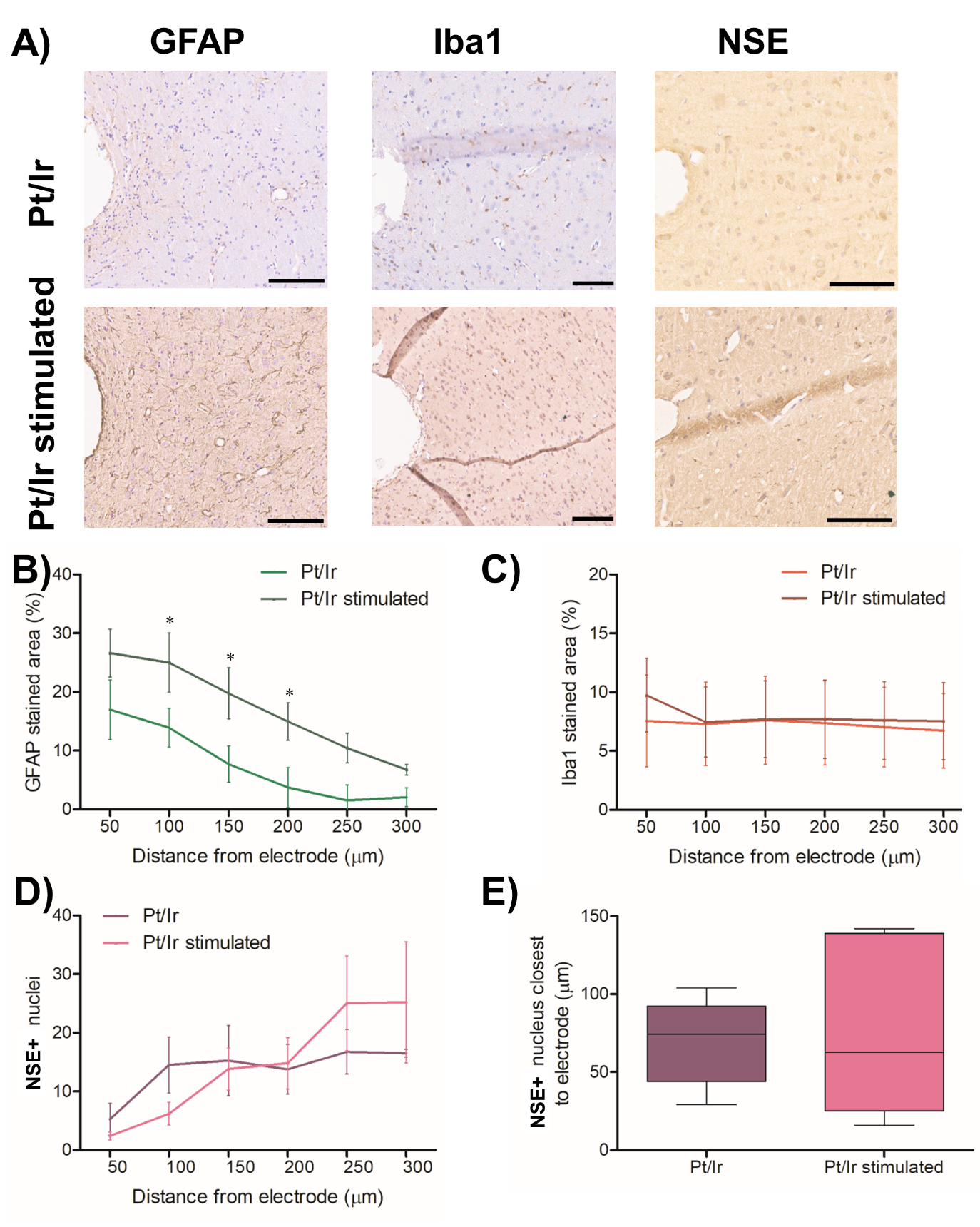
**

**Supplemental Figure S1: Foreign body response for PtIr and stimulated PtIr electrodes.**

**A)** Representative immunocytochemistry images of brain tissues implanted with pristine PtIr and stimulated PtIr electrodes (scale bar = 100μm). **B)** Staining area of the astrocytic protein glial fibrillary acidic protein (GFAP) (two-way repeated-measures ANOVA with Tukey post-hoc test: Distance: F(5, 45) = 12.47, P < 0.0001; Electrode: F(1, 45) = 8.56, P = 0.017; Interaction: F(5, 45) = 0.44, P = 0.82), **C)** microglia protein ionized calcium-binding adapter molecule 1 (Iba1) (Distance: F(5, 45) = 1.46, P = 0.22; Electrode: F(1, 45) = 0.02, P = 0.88; Interaction: F(5, 45) = 0.76, P = 0.58) and **D)** neuronal marker Fox-3/ Rbfox3/ Hexaribonucleotide Binding Protein-3 (NeuN) as a function of the distance from the electrode implantation site (Distance: F(5, 35) = 6.01, P = 0.0004; Electrode: F(1, 35) = 0.02, P = 0.88; Interaction: F(5, 35) = 1.55, P = 0.20). **E)** Mean distance to the nearest NeuN+ (mature neuron) nucleus from the electrode hole for each group (unpaired T-test: t(8) = 0.31, P = 0.77). Data are represented as mean ± SEM (n=6). * P < 0.05, ** P < 0.01, *** P < 0.001, **** P < 0.0001 (Data from Evers et al. 2022 reanalysed)

Here, paraffin embedded 3.5 *μ*m slices were stained for astrocytes (anti-GFAP polyclonal rabbit anti-GFAP, Z033401, DAKO, Denmark), microglia (anit-Iba1 goat anti-human plyclonal AIF1/Iba1, LS-B2402 LSBio, USA) and neurons (anti-NSE anti-rabbit polyclonal NSE, ab53025, Abcam, UK). Slides were scanned (ScanScope XT, Aperio Technologies, USA) and computationally analysed using custom developed code in MATLAB (Mathworks, USA). While both studies followed the same methodology and were performed in parallel, because two different staining methodologies have been used with different slice thicknesses, hydration levels and visualisation, a direct comparison between this data and Figure 3 in the main paper is not possible.
